# Awakening of Dormant Breast Cancer Cells in the Bone Marrow

**DOI:** 10.3390/cancers15113021

**Published:** 2023-06-01

**Authors:** Robert Wieder

**Affiliations:** Rutgers New Jersey Medical School and the Cancer Institute of New Jersey, 185 South Orange Avenue, MSB F671, Newark, NJ 07103, USA; wiederro@njms.rutgers.edu; Tel.: +1-973-972-4871

**Keywords:** dormancy, micrometastases, hematopoietic niche, bone marrow microenvironment, osteoblast niche, reawakening

## Abstract

**Simple Summary:**

Breast cancer cells travel via the bloodstream to the bone before the cancer is detectable in the breast. These disseminated cells are resistant to adjuvant chemotherapy and hormone therapy administered for the very purpose of eliminating them. They recur steadily for more than 20 years, resulting in incurable diseases. The bone marrow location, or niche, which normally provides a nest for blood-forming cells to enable them to generate blood for the entire lifetime of an individual, also protects these disseminated tumor cells and places them into a state of quiescence called dormancy. Dormant cancer cells can wake up from stimulation by life events, including a gradual increase in bone marrow fat cells and loss of estrogen with aging, inflammation, new blood vessel formation, trauma, surgery, abnormal blood clotting conditions, anxiety and depression. Many investigations have tested ways of killing disseminated cells or keeping them dormant, and some have entered clinical trials.

**Abstract:**

Up to 40% of patients with breast cancer (BC) have metastatic cells in the bone marrow (BM) at the initial diagnosis of localized disease. Despite definitive systemic adjuvant therapy, these cells survive in the BM microenvironment, enter a dormant state and recur stochastically for more than 20 years. Once they begin to proliferate, recurrent macrometastases are not curable, and patients generally succumb to their disease. Many potential mechanisms for initiating recurrence have been proposed, but no definitive predictive data have been generated. This manuscript reviews the proposed mechanisms that maintain BC cell dormancy in the BM microenvironment and discusses the data supporting specific mechanisms for recurrence. It addresses the well-described mechanisms of secretory senescence, inflammation, aging, adipogenic BM conversion, autophagy, systemic effects of trauma and surgery, sympathetic signaling, transient angiogenic bursts, hypercoagulable states, osteoclast activation, and epigenetic modifications of dormant cells. This review addresses proposed approaches for either eliminating micrometastases or maintaining a dormant state.

## 1. Introduction

More than 43,000 women in the US die from breast cancer (BC) every year, primarily from metastatic disease [1]. However, BC cells metastasize to the bone marrow (BM) before primary tumors can be detected and are found in the BM of 27–40% of newly diagnosed patients with localized primary disease [2,3]. Once they arrive at the BM, most micrometastases are killed by the hostile microenvironment; nevertheless, some of them enter a state of dormancy [4]. Dormant cells have cancer stem cell characteristics [5] and are resistant to adjuvant chemotherapy administered for the distinct purpose of eliminating them [6,7]. The metastatic niche contributes significantly to this resistance [8,9].

Metastatic breast cancer cells were first observed histologically in the bone marrow aspirates of breast cancer patients by Coombes et al. (1980), but the detection rate of 0.4% was highly inefficient [10]. In 2081, Dearnaley et al. [11] developed a technique for increasing the detection of breast cancer cells in the bone marrow by immunostaining bone marrow aspirate smears for epithelial membrane antigen (EMA), which is strongly expressed in breast carcinoma cells, but it is not present in normal bone marrow cells. They detected small numbers of carcinoma cells in bone marrow aspirate smears from patients with both early and metastatic cancers [11]. The cells were verified as epithelial and their presence was positively correlated with primary tumor size, intratumoral vascular invasion, positive lymph node status and estrogen receptor (ER)^−^ tumor status [12,13]. The presence of micrometastases was correlated with a shorter time to early relapse and shorter survival in a 28-month median follow-up study [13] and in a 76-month follow-up study [14]. In a 12.5-year follow-up study, the relapse-free survival and overall survival decreased and were found to be no longer significant, factoring in tumor size, lymph node status and vascular invasion [15]. However, Braun et al. demonstrated in a large study that the presence of bone marrow micrometastases at the time of diagnosis of localized disease is an independent negative prognostic indicator at 4 years [2], and that the effect is sustained at a 10-year follow-up [16]. Hormone receptor-positive (HR^+^) tumors continue to recur for more than 20 years in about 50% of mostly postmenopausal aging patients [3,16,17,18,19,20,21,22].

The BM is the most common site of metastasis for breast cancer, with BM metastases found in 73% of patients with breast carcinoma at autopsy, the highest of any cancer [23]. The BM presents a particularly restrictive environment for the outgrowth of metastatic cancer cells, killing most and inducing others to become dormant. The mechanisms responsible for the initial establishment and maintenance of dormancy in the BM are multifactorial and depend on microenvironmental signals, as well as on the genetic characteristics of the cancer cells themselves, including the hormone receptor status that directly affects some of the dormancy mechanisms. The factors contributing to their reawakening are also multifactorial and perhaps less well understood than the induction of the metastatic state and maintenance of quiescence.

Here, I review the data supporting mechanisms for homing of breast cancer cells to the bone marrow, data defining the hematopoietic stem cell (HSC) niches that the breast cancer cells usurp, and mechanisms controlling the dormancy and differentiation of HSCs by the BM niches. I present evidence from wide-spanning studies that define the mechanism responsible for maintaining cancer cells in the dormant state. Since the recurrence of dormant breast cancer cells is unpredictable and recurrent disease is incurable, I will outline data on the current understanding of the mechanisms that may play a role in dormancy reawakening and provide examples of approaches to eliminating dormant cells or maintaining them in a dormant state.

## 2. Breast Cancer Metastasis and Dormancy in the Bone Marrow

### 2.1. Hematogenous Transit of Cancer Cells to the BM HSC Niches

The homing of cancer cells to the BM is reported to approximate that of HSCs [24]. It is mediated through the C-X-C motif chemokine receptor 4 (CXCR4), the receptor for stromal cell-derived factor-1/C-X-C motif chemokine 12 (SDF-1/CXC ligand (CXCL)12) [25,26,27] and through annexin II [28], which is required for hematopoietic stem cell transplants [29]. Annexin II serves as an anchor for CXCL12 to localize HSCs [30] and cancer cells [31] to the niche. Homing also involves cadherin-11 [32,33], osteopontin [34], connective tissue growth factor (CTGF) [34], and Runt-related transcription factor 2 (RUNX2) [35]. Cadherin-11 induces the expression of the gamma-carboxyglutamic acid (Gla) domain-containing protein 6 (GAS6) receptors AXL tyrosine kinase (AXL), skywalker (Sky) and Mer proto-oncogene tyrosine kinase (Mer), which induce dormancy in hematopoietic stem cells [36]. 

Disseminated tumor cells (DTCs) in the BM interact with a wide array of cell types, proteins, proteoglycans, growth factors and cytokines endemic to the hematopoietic microenvironment, which, together with its biophysical and bioenergetic characteristics, regulate dormancy and participate in reawakening [37]. The BM hematopoietic microenvironment is made up of a complex network of cells consisting of mesenchymal cells of different lineages and degrees of stemness, osteogenic cells, chondrocytes, adipocytes, neuroglial cells, hematopoietic lineage cells, including megakaryocytes and macrophages, cells of the sympathetic nervous system and a network of endothelial cells that include cells lining the sinusoids, arterioles and transition zones [38]. These data indicate the existence of two primary niches that maintain HSC dormancy, the preosteoblast endosteal-lining niche [39] and a more centrally located parasinusoidal endothelial niche, with some endothelial cells also residing near the endosteum [40]. The two positions have been reconciled by arguments that the endosteal niche is also vascularized [41], that the niches are in close proximity and that, because of the newly recognized heterogeneity in the HSC population gleaned from single-cell sequencing, different niches may provide support or imprint distinct HSC states for differently primed HSCs [38,42,43,44,45]. 

**Figure 1:**
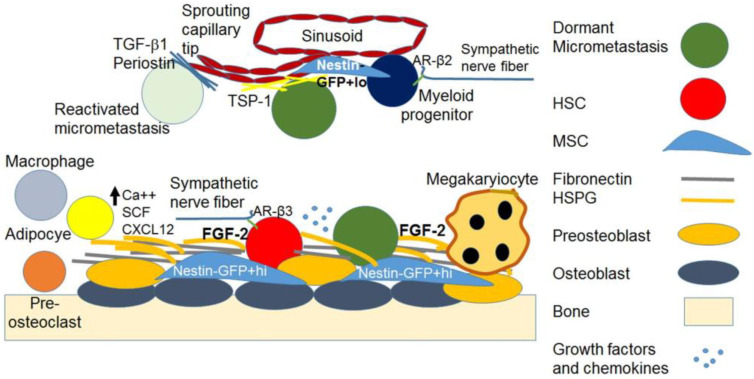
**The bone marrow hematopoietic and dormant metastatic breast cancer niches.** The image depicts simplified representations of the endosteal and parasinusoidal niche containing the HSC, the dormant micrometastasis, and the supporting cellular, structural and soluble elements. Growth factors and chemokines listed in the text are depicted as a cluster of light blue dots.

The endosteal niches consist of osteoblasts embedded in the bone matrix and preosteoblasts that are in contact with HSCs and Nestin-GFP^+hi^ mesenchymal stem cells (MSCs), which regulate HSC maintenance. Macrophages also inhabit the endosteum and help maintain the HSC niche. The sympathetic nervous system maintains HSCs through nerve fibers in the endosteum. Structural elements of the endosteum also support the dormancy of HSCs, including fibronectin and heparan sulfate proteoglycans (HSPGs) that serve as reservoirs for fibroblast growth factor (FGF)-2, which is important in HSC dormancy (Figure 1). I have discussed these members of the HSC niche below.

The true, rare, dormant and undifferentiated HSCs reside on preosteoblasts in the endosteal stromal and preosteoblast stem cell niches, which support their quiescence and self-renewal [46,47]. Once they acquire a lineage marker, they move to the sinusoidal endothelium where they can be tapped to differentiate into myeloid lineages [46,47]. 

Only immature osteoblasts support the dormancy of HSCs [48], whereas ossified, alkaline phosphatase (ALP)-expressing differentiated osteoblasts no longer support HSCs [49,50,51,52]. Signaling through parathyroid hormone (PTH) and interleukin (IL)-6, and adhesion to vascular cell adhesion molecule (VCAM)-1 and activated leukocyte cell adhesion molecule (CD166) in osteoblast precursors is necessary for appropriate HSC maintenance and lymphoid differentiation [49,50,51]. An array of adhesion molecules, growth factors and chemokines interact to maintain HSCs in a quiescent state in their niche and to mobilize them as needed for recruitment to the vascular niche [47]. These include Angiopoietin-1, Tie-2 and N-cadherin, which are associated with quiescence and cell-cycle control and adhesion molecules, including very late antigen (VLA)-4, lymphocyte function-associated (LFA)-1, osteopontin and integrins [47]. These molecules, along with CXCL12 (SDF-1) on stromal cells, its receptor CXCR-4 on HSCs, matrix metalloproteinase (MMP)-9, MMP-2 and stem cell factor (Kit Ligand) induced by granulocyte-colony stimulating factor (G-CSF), SDF-1, FGF-4, vascular endothelial growth factor (VEGF) and placental growth factor (PLGF), are required for HSC recruitment and mobilization [47]. Calcium in the endosteal niche is also important for the support of HSCs [53]. HSC maintenance and quiescence, hematopoietic reconstitution and protection from aging-associated DNA damage also depend on interactions with periostin via integrin αv and inhibition of focal adhesion kinase (FAK)/phosphoinositide 3-kinase (PI3K)/Ak strain transforming (AKT) signaling, leading to an increase in p27^Kip1^ [54].

The sympathetic nervous system β2 adrenergic signaling releases HSCs from the osteoblastic niche by inducing the transcription of the calcium-regulating hormone vitamin D receptor (VDR) and its downstream gene receptor activator of nuclear factor kappa-Β ligand (RANKL), an effect stabilized by 1,25 dihydroxy-vitamin D_3_ [55]. While the mean extracellular calcium ion concentration [Ca^++^]_e_ in the BM is 1.0 ± 0.54 mM, which is not significantly different from that in the blood serum, the [Ca^++^] in the location of the HSCs is 1.5 ± 0.57 mM and significantly increases with aging to support myeloid clonal expansion [56].

Aging and senescence of HSC niches result in changes in the character, makeup, location and differentiation dynamics of HSCs by regulating niche remodeling [42]. Aging induces a functional decrease in adrenergic receptor (AR)-β3 activation and increases AR-β2 (ADRB2) activation [42,47,57]. This induces HSC migration from osteoblasts to the sinusoids, skewing hematopoiesis towards myeloid differentiation, megakaryocyte and platelet production, and decreases endosteal cells, resulting in lymphoid deficiency [42,47,57]. Aging stroma results in a decrease in endosteal and in an increase in sinusoidal Nestin-GFP^+hi^ cells, events that also play a role in the myeloid skewing of hematopoiesis. The movement of Nestin-GFP^+hi^ cells, which give rise to all mesenchymal lineages, including osteoblasts and adipocytes, to the sinusoidal space induces the migration of Jagged-stained cells and associated HSCs to the sinusoids [42,58]. 

There is a global decrease in osteoblastogenesis and an increase in adipocytogenesis with aging, ovariectomy and other causes of osteoporosis or disease [59,60,61,62]. Indeed, reinforcing the role of adipocytes in the loss of dormancy, perivascular cells express an adipocyte-skewed expression profile that promotes proliferation [63]. Mature adipocytes activate extracellular receptor kinase (ERK or MAP kinase) signaling in multiple myeloma cells [64]. Overall vascular density and leakiness increase and sinusoidal notch receptor (Notch) activity decreases with aging [42]. These effects are accompanied by an overall increase in inflammation and secretory senescence, characterized by increased levels of inflammatory cytokines IL-1, IL-3, IL-6, tumor necrosis factor (TNF)α, interferon (INF)γ and transforming growth factor (TGF)-β, which regulate myeloid skewing [42].

The role of osteoblasts in the maintenance of the undifferentiated quiescent HSC state is supported by experiments in which a preosteoblast knockout was sufficient to induce leukemia [65,66,67], and its replacement restored normal hematopoiesis [68]. Many adhesion molecules, growth factors and chemokines interact to maintain HSCs quiescence in their niche and mobilize them to the vascular niche as needed [47,49,50,51]. These include bone morphogenic proteins (BMPs), TGF-β2 [46] and FGF-2 [69,70,71]. FGF-2 is important for the maintenance [69], self-renewal [70,71,72] and myeloid differentiation of HSCs [73,74], but blocks erythroid and B lymphocyte differentiation [75] and myeloid differentiation at high concentrations [76].

### 2.2. The Metastatic BM Niches and DTC Dormancy Signaling

Most metastases die in the hostile microenvironment of the BM, but some survive and enter a state of dormancy [77]. Early arriving DTCs that survive the microenvironmental effects generate a metastatic niche, combine with late metastases and potentially remain quiescent or in an ultra-slow cycling mesenchymal state in the HSC niche [78,79,80] for periods lasting up to decades [46,81]. 

The fate of the cancer cells depends on the opposing efforts of the microenvironment and the cancer cells. The microenvironment endeavors to suppress the cancer cells, while the cancer cells exert their efforts to turn MSCs and fibroblasts into cancer-promoting cells [82]. The cancer cells attempt to generate a pre-metastatic niche with the potential to support cancer cell colonization through the modulation of MSC through microvesicles [83] and through the secretion of inflammatory cytokines, which recruit BM-derived cells and form an inflammatory milieu that supports colonization [84]. They also secrete factors that enhance bone resorption, such as lysyl oxidase (LOX), a collagen crosslinking enzyme produced primarily by hypoxic ER^−^ cancer cells, parathyroid hormone-related peptide (PTHrP), osteopontin (OPN) and CC-chemokine ligand (CCL)-2, directly promoting bone resorption and extracellular matrix (ECM) remodeling, making the niche more permissive to DTCs [85]. However, metastatic cancer cells also process structural proteins such as fibronectin in the microenvironment, which in turn induce quiescence and survival signaling in the cancer cells [81]. Ultimately, the scarcity of micrometastases is the most likely reason why the cancer cells’ attempts at modifying the niche to promote cancer growth are overwhelmed by the collective suppressive effects of the cellular, structural and soluble factors of the niche [82]. DTCs in the BM interact with a wide array of cell types, proteins, proteoglycans, growth factors and cytokines endemic to the hematopoietic microenvironment, which, together with their biophysical and bioenergetic characteristics, regulate dormancy and eventually participate in reawakening [37].

Cancer cell dormancy in a niche can be considered an adaptive state, guided by the thermodynamics of local energy minima, mechanical confinement [86] and hypoxia [87]. The preparation of the metastatic niche may be aided by the presence of VEGF receptor (VEGFR)1^+^ hematopoietic progenitor cells [88], as well as the deposition of extracellular matrix by micrometastases [89]. In the metastatic HSC niche, BC cells interact with cellular, structural and soluble factors to initiate dormancy [88,90,91,92,93,94], including NG2^+^/Nestin^+^ mesenchymal stem cell-initiated TGF-β2 and BMP7 signaling [95]. Cells include MSCs, fibroblasts, osteoblasts, adipocytes, Nestin^+^ endothelial cells, T-cells and macrophages. Structural factors include fibronectin, p-selectin, thrombospondin and HSPG. Soluble factors include Bmp4, Bmp6, Bmp7, kit ligand, TGF-β1 and β2; Dickkopf-related protein 1 (Dkk1) and Dkk3, thombospondin2 (Thbs2) found in the BM secretome [96,97] and FGF-2 [88,90,91,92,93,98]. FGF-2 is deposited on stromal HSPGs [99], which are needed for FGFR dimerization [100] and are able to induce dormancy [101]. Fibronectin, an integral element of the endosteum [102], also induces dormancy [103,104] and can prevent transformation [105]. Signaling initiated by structural proteins in the BM also depends on their variable structural organization [103], tensile strength and mechanical signaling [106].

Dormant BM micrometastases have marked genetic heterogeneity [107,108]. However, most express the hyaluronan receptor CD44 and about half of the cells are also CD24^−^, identifying them as having tumor-initiating characteristics that enable them to regrow into breast tumors [109] and express a stem cell program [110]. Signaling initiated by osteoblast interactions seems to maintain tumor-initiating properties in DTCs [111]. 

Maintenance of stemness was also reported to be mediated by FGFR2 [112] and FGFR-initiated signaling through Akt/Sry-related HMG-box (Sox)2 [113], stem cell-like chromatin rearrangement through the inhibition of cyclin-dependent kinase (CDK)4/6 and upregulation of programmed cell death protein (PD)-1 [114,115], protecting dormant cells from immune elimination. A key niche factor for inducing and maintaining ER^+^ BC dormancy is FGF-2, which is synthesized and exported by stromal fibroblasts and subsequently deposited on HSPGs overlying the stroma [98,99]. FGF-2 also plays a key role in the maintenance of HSC dormancy, as noted above [112,113]. HSPGs are necessary for the dimerization of FGF receptors [100] and for maintenance [116], multipotency [117] and osteogenic differentiation of MSCs [118]. Similarly to its role in HSCs, FGF-2 also supports the dormancy of hormone receptor-positive BC cells through dual signaling by FGF-2-induced re-expression of integrin α5β1, which binds to microenvironmental fibronectin [98,119,120,121]. FGF-2 inhibits breast cancer cell proliferation and response to chemotherapy through the activation of ERK [122], phosphoinositol-3 kinase (PI3K) [98,119] and intracellular TGF-β-mediated upregulation of cyclin-dependent kinase inhibitors p21^Waf1^, p27^Kip1^ and 15^INK4b^ [123,124], inactivation of CDK2 and CDK4, and dephosphorylation of retinoblastoma protein (Rb) [123], mechanisms which have been confirmed in subsequent investigations [114,115]. Dormant cells maintain a characteristic, large, spread out, non-motile epithelial phenotype through dual FGF-2 and fibronectin-activated integrin α5β1 signaling [120]. The phenotype is due to the inhibition of Ras homology family member (Rho)A by the Rho GTPase activating protein (Gap) 26 (GRAF), a resulting cortical actin rearrangement and an omnidirectional activation of FAK [120]. Fibronectin, which is deposited abundantly in the BM microenvironment [102], suppresses the malignant phenotype [103,105] and collaborates with integrin α5β1 to establish the premetastatic niche [81]. ER^−^ cells are not inhibited by FGF-2 and do not utilize the fibronectin-FGF-2 dual signaling model to become dormant [98]. However, stromal MSCs do inhibit ER^−^ BC cells in a transwell co-culture model through the transfer of micro (mi)RNAs 127, -197, -222 and -223 [125,126,127] or SDF-1a [128] and decrease in CXCL12 levels [126]. This interaction is reciprocal, as metastatic breast cancer cells in the bone marrow microenvironment participate in remodeling the niche to sustain their dormancy [129]. In addition to its role in BC [130], FGF-2 also promotes stemness in benign prostate cells [112] and pancreatic cancer [113], and induces dormancy in ER^+^ BC cells [98]. Quiescent cancer micrometastases express dormancy signatures [78,79,131] similar to those modulating normal stem cell quiescence [78,80]. FGF-2 acts in concert with structural proteins in the microenvironment, where dormant micrometastases become anchored in place by binding to microenvironmental proteins and cellular components [98,104].

The MSC niche, the vascular niche and the immune niche provide support for metastatic BC cell survival and dormancy through a variety of mechanisms (Table 1) [46]. Metastatic cells survive in the BM hematopoietic microenvironment in close proximity to stromal cells in the endosteum, where they occupy the hematopoietic stem cell niche [132], as well as in the perivascular endothelium [8]. The mesenchymal stem cell niche activates multiple signaling pathways in dormant cells via receptors Mer tyrosine kinase (MERTK), AXL and its ligand GAS6 [36,133], TGF-β2 through TGF-β receptor 3 and BMP receptor 2 via SMAD family members (SMAD)1 and 5, basic helix–loop–helix family member E41 (DEC2), the metastasis suppressor gene N-Myc downstream-regulated (NDRG)1, BMP4 and 7 through BMP receptor (R)2, activated p38 MAP kinase (p38) [90] and its downstream target mitogen- and stress-activated kinase 1 (MSK1) [134], activin receptor-like kinase (Alk)5 [135], inactivated ERK, and cyclin-dependent kinase inhibitors p21^Waf1^ and p27^Kip1^ [46,94,136]. Other stem cell niche signals also regulate dormancy [88,90,91,93], potentially by antagonizing oncogene signaling [92]. 

The BM is a hypoxic environment [137], a factor implicated in the induction of dormancy [138] by repressing the leukemia inhibitory factor (LIF)-signal transducer and activator of transcription (STAT)3. Primary tumor hypoxia presets primary tumor cells with a program supporting dormancy, which manifests after dissemination to the metastatic niche [139]. Redox signaling in the microenvironment also generates enabling conditions for dormancy signaling, remodeling of the microenvironment, reprogramming of DTC dormancy signaling and maintenance of epithelial-mesenchymal transition (EMT) and stemness [140]. Microenvironmental redox signaling also generates therapeutic resistance in dormant cells through vigorous induction of antioxidant mechanisms to counter cytotoxin-induced oxidative stress, apoptosis, autophagy and oncogenic bypass signaling [140]. Conversely, redox signaling can also play a role in reawakening [140].

Other factors involved in inducing dormancy are retinoic acid, leukemia inhibitory factor (LIF), wingless-related integration site (Wnt) family members, miR-126 and DNA methylation (reviewed by Risson et al., 2020) [94]. Stroma also produces exosomes overexpressing miR-23b [125] or other miRNAs [126,127] that are transferred to DTCs, which also endow dormancy signaling [96,125,126,141]. Wnt5a non-canonical Wnt signaling induces dormancy in prostate cancer cells in vitro and in vivo in the BM osteoblast niche in a reversible manner via receptor tyrosine kinase-like orphan receptor 2 (ROR2)-activation of siah E3 ubiquitin protein ligase 2 (SIAH2) expression, which represses canonical Wnt/β-catenin tumor stem cell and tumor progression signaling [142]. Wnt family members regulate MSCs in their niche in the BM stroma, where Wnt5a localizes with cells that are leucocyte common antigen (CD45)^+^, which are transmembrane protein tyrosine phosphatases located on most hematopoietic cells, and CD45^−^ mesenchymal stem cell marker (STRO-1)^+^ mesenchymal progenitor cells, whereas canonical Wnt is associated with the underlying stroma matrix [143]. Wnt3a expands the pool of MSCs capable of generating colony-forming unit-fibroblasts (CFU-F) and CFU-osteoblasts (O), whereas Wnt5a maintains the pool of cell numbers, CFU-Fs and CFU-Os, suggesting a potential dual role of Wnt5a in the maintenance of MSCs in the BM and in enhancing osteogenesis [143].

The BM microenvironment has a low oxygen tension, which predisposes cancer cells to fuse with MSCs and other cells in the microenvironment, and, in fact, the fusion of BC cells with MSCs can induce dormancy [144], as well as a spectrum of other functions in BC cells [145]. One study suggests that cancer cells cannibalize stromal mesenchymal cells to become dormant [146]. The BM interstitial pH ranges from 6.7 to 7.5 (7.0–7.3 within a 10% to 90% confidence interval), with a mean value of 7.1, which is slightly more acidic than the blood serum that is close to 7.4 [56]. The BM oxygen tension is <1–6% (~7 mm Hg–43 mm Hg), as compared to most normal tissues of 2% to 9% (14–65 mm Hg) [87]. This hypoxic, acidic microenvironment generates a redox imbalance, which, combined with a slightly hypertonic medium and TGF-β and BMP signaling, is sufficient to drive cancer cells to a gene expression pattern with characteristic features of the dormancy signature [147].

In the osteoblast niche, data suggest that metastatic malignant cells usurp the HSC niche to create an abnormal niche that is unable to support normal hematopoiesis [63,132,136,148]. BC cells with a stem cell phenotype compete with HSCs in the endosteal niche and remain dormant in a Notch-dependent manner by spindle-shaped N-cadherin^+^ CD45^−^ osteoblasts (SNO cells) [149]. Micrometastases survive in the HSC niche close to endosteal stromal cells [132], where preosteoblasts support their survival and chemoresistance [9], partially mediated by Jagged1 [150]. As noted, it is the preosteoblasts that likely support dormancy [48], but as osteoblasts differentiate, they connect with cancer cells through gap junctions, increase their intracellular calcium levels and potentially promote colonization [53]. Jagged-1/Notch signaling regulates tumor stem cell development, epithelial-to-mesenchymal transition, and immune cell homeostasis during minimal residual disease, and plays a role in the recurrence of minimal residual disease in primary tumors [151]. However, some osteoblasts in the HSC niche become “educated” by arriving cancer cells to support the dormant state [152]. These “educated” osteoblasts express RUNX2/osteocalcin (OCN)/OPN, are negative for IL-6 and α-smooth muscle actin (αSMA), and have new properties where they acquire the capacity to suppress both triple-negative and ER^+^ breast cancer cell proliferation [153]. They increase cancer cell p21^Waf1^ expression [152], regulate ERK 1 and 2 signaling and inhibit S-phase entry [153]. These effects are mediated by the secretion of small extracellular vesicles enriched for miR-148a-3p [153]. These data underscore the reciprocal relationship between the cancer cells educating the metastatic microenvironment in the premetastatic and dormancy niches, and the dormancy-endowing effects of the niche on cancer cells. 

In the vascular niche, non-sprouting endothelial cells produce ECM components such as thrombospondin-1 (TSP1), which may induce dormancy [46]. The endothelial Duffy antigen receptor for chemokines (DARC) may induce dormancy in cancer cells by binding to the metastasis suppressor cluster of differentiation 82 (KAI1), inhibiting proliferation through p21^Waf1^ and downregulating T-Box transcription factor 2 (TBX2) [154]. Signaling mechanisms associated with micrometastasis dormancy include von Willebrand factor (vWF) [8], VCAM1 [8], CXCL 1 and 2 [155], BMP7 [90], TGFβ-2 [91], canonical nuclear factor (NF)κB combined with ER signaling in ER^+^ BC cells [156], nuclear receptor subfamily 2 group F member (NR2F)1 [93] and zing finger protein (ZFP)281 [157]. Dormant stem cell signaling through phosphatase and tensin homolog (PTEN) maintains a dominant role in tumorigenic stimuli [92]. Perivascular periaxin (Prx)1^+^ MSCs express CXCL12 and maintain quiescence and chemoresistance of leukemic stem cells, in contrast to their effects on HSCs, suggesting a more complex mechanism that differentiates the roles of the endothelial niche in malignant vs. normal hematopoietic stem cell maintenance [158].

However, signaling in the vascular endothelial niche is not all dormancy-inducing in malignant cells [159]. Analogous to the case of hematopoietic stem cells that receive pro-differentiating signals once they translocate to the endothelial niche, cancer cell micrometastases can receive context-specific proliferative signals in their interactions with endothelial cell tips mediated through TGF-β1 and periostin [159]. Indeed, the effects of periostin appear to be context-specific, as some of its effects on HSC are linked to stem cell maintenance in the endosteal niche, as noted above [54]. Endothelial cells can promote a stem-like phenotype in some solid tumor cancer cells through the activation of the hedgehog pathway through Gli-1 [160]. Gli-1 expression is high in breast cancer and contributes to therapeutic resistance in both ER^+^ [161] and ER^−^ BC cells [162,163] through Wnt signaling [162]. Endothelial cell L1 cell adhesion molecule (L1CAM) ligands may induce the proliferation of L1CAM^+^ DTCs [94]. Endothelial leukocyte adhesion molecule 1 (E-selectin) signaling in endothelial cells induces a non-canonical mesenchymal–epithelial transition (MET) phenotype in cancer cells, which begin to express EpCam and cytokeratin 14 (CK14) while continuing to express mesenchymal gene expression factors including snail family transcriptional repressor (Snail) 1/2, twist family bHLH transcription factor (Twist) 1/2, zinc finger E-box binding homeobox (Zeb) 1/2 and cancer stem cell marker Sox 2/9 [164]. These programs permit the regrowth of dormant micrometastases [164]. The conditions for the recurrence of cells expressing mesenchymal programs are discussed below [121]. Quiescent DTCs in the BM lack the epithelial marker E-cadherin [165], but do not undergo a phenotypic appearance of EMT [166]. This is corroborated by our in vitro data supporting a model for continued mesenchymal signaling in dormant cells with an apparent epithelial phenotype [120,121], as discussed below. However, once these cells are stimulated to undergo MET, they begin to proliferate once again. Micrometastatic sites can serve as launching pads for colonization and re-metastasis [167].

The immune niche contains macrophages and CD4^+^ and CD8^+^ cells that may induce dormancy [166] through INFγ [46]. Quiescent cancer cells in distant organs that have tumor-initiating capacity express DKK-1, which inhibits Wnt, enhancing the downregulation of Natural Killer (NK) cell activators and death ligands, and evading killing by NK cells [168]. In addition to homing, SDF-1/CXCR4 may promote survival through Src through Akt and TNF resistance through TNF-related apoptosis-inducing ligand (TRAIL) [169]. Secretion of SDF-1α by BM MSCs may maintain quiescence in breast cancer micrometastases by downregulating the truncated neurokinin receptor-1 (NK1R-Tr) expression [128].

Signaling intrinsic to the cancer cell is also likely to contribute to the dormant state by expressing metastasis suppressor genes that contribute to dormancy without affecting the growth of cells in the originating primary tumor [170]. The tyrosine kinase receptor TIE2, which induces dormancy in hematopoietic stem cells, also induces cell cycle arrest in breast cancer cells through CDK inhibitors *CDKN1A* (p21^Waf1^) and *CDKN1B* (p27^Kip1^) in vitro, decreases osteolytic metastases and response to antimetabolites in mice, and is associated with delayed time to metastasis in breast cancer patients [171]. Expression of the metastasis suppressor genes KISS-1, metastasis suppressor Kangai-1 (KAI1), mitogen-activated protein kinase (MKK)4/7 and NM23 nucleoside diphosphate kinase 1 (Nm23-H1) by cancer cells also promotes tumor dormancy at the metastatic site [170]. Signaling through indoleamine 2,3-dioxygenase 1 (IDO1) through the mammalian target of rapamycin (mTOR) and control of nonderepressible-2 kinase has been linked to cellular quiescence [172]. IDO1, which is a heme-containing enzyme that mediates the rate-limiting step in the metabolism of l-tryptophan to kynurenine, has been explored as a potential immunotherapeutic target in oncology [173]. An inhibitor of this pathway has been found to have an acceptable toxicological spectrum in animal studies [173].

Other mechanisms of inducing reversible dormancy functions through epigenetic modifications, such as repressive histones [174] or the downregulation of suppressor gene promoter methylation enzymes [175], have been explored. These effects are analogous to evolutionary mechanisms that ensure the survival of organisms in environmentally disadvantageous circumstances [176]. They can also originate in the primary tumor, where epigenetic modifications in some of the cells enable them to enter dormancy in a distant microenvironment by cancer-associated fibroblasts (CAF) with altered p53 functions [177]. In tumor cells, the downregulation of DNA methyltransferase (DNMT)1 expression results in silencing a transcription network regulating the G1-S transition, including forkhead box (FOX)M1, FOXD, FOXL, early growth response (EGR)1/2/3, peroxisome proliferator-activated receptor (PPAR)γ, ETS Like-1 protein Elk-1 (ELK1) and Jun family members [78,176]. However, the dormancy-associated genes p53, DEC2, nuclear receptor subfamily 2 group F member (NR2F)1 and retinoic acid receptor (RAR)β, which are often silenced in proliferating cancer, are upregulated in dormant cells [78,176]. NR2F1 and RARβ together direct the removal of acetyl groups from histone H3 by histone deacetylases (HDACs) and are associated with dormant DTCs in patients [20,93,178]. In contrast, NR2F1 induces the methylation of H3 residues histone H3 (H3)K4, H3K9 and H3K27 and decreases the expression of growth-promoting SOX9 [176]. Histone H4 methylation is necessary for breast cancer dormancy in the lungs [179]. Epigenetics also affect dormancy and proliferation by governing the processing of coding mRNA alternative isoforms and non-coding RNAs, including micro-RNAs and long non-coding RNAs [180,181]. 

The overall effect of the metastatic microenvironment is to impose a reversible state of dormancy on the microscopic disseminated tumor-initiating cells. This effect is mediated through cancer cell interactions with structural, soluble, cellular and biophysical elements of the microenvironment that initiate signaling through a variety of receptors and sensors outlined above in order to change gene expression and phenotypic patterns to induce a dormant, cytotoxin-resistant state.

**Table 1:** Mechanisms of breast cancer dormancy in the bone marrow.

	Vehicle	Signaling	References
Endosteal niche	MSCs	MERTK, AXL, TGFβR3, BMPR2, Alk5, NDRG1, ERK, p38, p21^WAF1^, p27^Kip1^, 15^INK4b^, PI3K, RhoA/GRAF, integrin α5β1, FGF-2, HSPG, fibronectin	[36,46,81,88,90,91,93,94,98,99,100,102,103,105,114,115,119,120,121,122,123,124,125,126,127,128,129,130,131,132,133,134,135,136]
		Inhibition of oncogene signaling	[92]
		Non-canonical Wnt5a signaling, SIAH2, repression of β-catenin, LIF, RA	[94,142,143]
	Hypoxia, acidic pH	LIF, STAT3, TGFβ, BMP signaling	[87,139,147]
	Redox signaling		[140]
	Exosomes	miR-23b, -126, 127, -148a, -3p -197, -222, -223	[94,95,125,126,127,141,153]
	Fusion with and cannibalizing MSCs	SDF-1a, decreased CXCL12	[126,128,144,146]
	Microenvironmental remodeling		[129]
	Preosteoblasts, SNO cells	Notch, Jagged1	[48,149,150]
Vascular niche	Endothelial cells	TSP1	[46]
		DARC, KAI1, p21^Waf1^, downregulated TBX2	[154]
		vWF, VCAM1, CXCL 1 and 2, BMP7, TGFβ-2, NFκB combined with ER in ER^+^ BC, NR2F1, ZFP281, PTEN	[8,91,92,93,155,156,157]
	Prx1^+^ MSCs	CXCL12	[158]
Immune niche	CD4^+^ and CD8^+^ cells	INFγ	[46,166]
	NK cells	DKK-1, inhibited canonical Wnt	[168]
	SDF-1/CXCR4	Src, Akt, TRAIL, downregulated NK1R-Tr	[128,169]
Cancer cell-intrinsic effects	TIE2	p21^Waf1^, p27^Kip1^	[170,171]
		KAI1, MKK4/7, Nm23-H1	[170]
	IDO1	mTOR	[172]
Epigenetics	Repressive histones	altered p53 functions	[174,175,177]
	downregulation of suppressor gene promoter methylation enzymes		
	downregulation of DNMT1	silencing of a transcription network FOXM1, FOXD, FOXL EGR1/2/3, PPARγ, ELK1, Jun family upregulating p53, DEC2, NR2F1, RARβ	[78,176]
	NR2F1, RARβ	removal of acetyl groups from histone H3, HDACs	[20,93,178]
	NR2F1	induced methylation of H3 residues H3K4, H3K9, H3K27, decreased expression of growth-promoting SOX9	[176]
		processing alternative coding mRNA isoforms, non-coding RNAs, miRNAs, lnRNAs	[180,181]

## 3. Reawakening of Dormant Cancer Cell

### 3.1. Clinical Cancer Variables Associated with Recurrence

Studies using a variety of databases have reported variables associated with the recurrence patterns of dormant disease in patients with localized BC. Patient variables include age, race and body mass index; cancer variables include stage, grade and proliferative status, hormone receptor and human epidermal growth factor receptor (Her)2 status; and treatment variables include adjuvant chemo-, hormone- and biotherapy. In one analysis, ER^+^/Her2^−^ lymph node-negative patients with smaller primary tumors of 1–10 mm diameter had an 88% long-term distant recurrence-free interval (DRFI) compared to 77% in patients with 10–20 mm diameter tumors [182]. Patients with tumor grade 1 had an 81% DRFI vs. 77% in patients with tumor grade 2 and 65% in patients with tumor grade 3 [182]. A long-term tamoxifen benefit was observed among patients with larger tumors and higher grades, and in patients who were PR^+^ [182]. In a meta-analysis of 88 trials of women with ER^+^ BC who were disease-free after 5 years of endocrine therapy, the patients had steady recurrence rates from 5 to 20 years. The risk of recurrence correlated with the original tumor/node (TN) status, PR^+^, Her2, grade and Ki-67, with risks ranging from 10 to 41%, depending on the TN status and tumor grade [183]. 

Of the listed variables that affect the reawakening of dormant tumors of stages I–III in patients who received therapy with curative intent, the tumor hormone receptor status allocated them into two different recurrence categories. Stage I–III BC patients were 78.4% ER^+^/21.6% ER^−^ and 68.1% PR^+^/31.9% PR^−^ [184], and stage IV BC patients were 71.3% ER^+^/28.7% ER^−^ and 68.0% PR^+^/32% PR^−^, according to the data from the SEER database [185]. When comparing women with triple-negative localized BC to women diagnosed with other types of local BC, the hormone-negative group showed an increased hazard ratio of distant recurrence of 2.6 within 5 years of diagnosis but not thereafter [17]. The risk of distant recurrence of triple-negative BC peaks at approximately 3 years and declines rapidly thereafter compared to other groups, whereas the recurrence risk appears constant over a median follow-up of 8.1 years [17].

In one study, patients had a 19.8% rate of first recurrence in the first 10 years after diagnosis, with 72.5% being distant metastases, with the highest risk occurring at 3.9% in the second year after diagnosis [186]. The prognostic factors for the first recurrence were age < 40 years, tumor size > 2 cm, tumor grade II/III, positive lymph nodes, multifocality, and no chemotherapy. In this study, in the first 10 years after diagnosis, the hazard ratio for distant metastatic recurrences for ER/PR^+^ tumors was 0.56 by univariate analysis compared to that of ER/PR^−^ tumors [186].

A seminal study of the recurrence of BC after diagnosis of local disease demonstrates that the probability of being free of distant recurrence in triple-negative BCs decreases rapidly between 1 and 5 years after diagnosis and then settles at a constant rate [17]. This pattern is in stark contrast to that of ER^+^ cancers, which show a steady decline in the status of being free of distant recurrence for 15 years [17]. Another study reports that the annual hazard of recurrence for all of the patients in their database is highest during the first 5 years (10.4%), with a peak between years 1 and 2 (15.2%) [187]. The study also notes that patients with ER^+^ disease have a lower annualized hazard ratio of recurrence compared to ER^−^ disease in the first 5 years (9.9% vs. 11.5%; *p* = 0.01) [187]. This pattern switches after 5 years, with ER^+^ patients having statistically significantly higher hazard ratios for recurrence than ER^−^ patients during every 5-year interval up to 25 years after diagnosis. The curves for the recurrence-free intervals cross at 48% at 8.5 years after diagnosis, whereas disease-free intervals for ER^+^ patients continue to decrease to about 32% at 26 years after diagnosis, while the intervals for ER^−^ patients flatten out at about 38% at 26 years [187]. Survival curves have similar characteristics, where the ER^+^ and ER^−^ curves intersect at about 50% at 14 years, with survival at 26 years being 28% for ER^+^ patients and 33% for ER^−^ patients [187]. The crossover for the hazard ratios for the survival of ER^+^ and ER^−^ patients is reported to be 8 years [18]. The patterns of survival are similar to data from a different database and have a distinct racial impact [184]. The crossover point for survival is about 55%, occurring at 15.1 years for Caucasians and at 13 years for African American (AA) patients [184]. The 26-year survival rate of ER^+^ patients is 40% for Caucasians, 38% for AA patients, 47% for ER^−^ Caucasian and 41% for ER^−^ AA patients [184]. The fact that the hazard ratios for BC death peak between 2 and 3 years and then decline rapidly and that this peak is much greater in ER^−^ than in ER^+^ tumors suggest that the processes governing early and late recurrence events and processes responsible for dormancy and recurrence of ER^+^ and ER^−^ cancers are different [18].

The probabilities of relapse can also be modeled from the patient and tumor features at the time of diagnosis using artificial intelligence and deep learning. By using a small number of features in a small study of 256 patients, a deep learning model was able to reach an accuracy of 77.50% and 80.39% and a sensitivity of 92.31% and 95.83% for predicting recurrence within 5 years and 10 years, respectively [188]. In a larger study of over 13,000 patients, a machine learning neural network program used 325 elements of clinical data from the time of diagnosis to identify 32 features to predict BC recurrence in real time [189]. The concordance index was 0.92 for the training data set and 0.89 for the validation and test data sets, with the area under the curve receiver operating characteristics performance measurements being 0.9 at the 2-year point and 0.91 at the 5- and 7-year points, values which are considered outstanding [189].

### 3.2. Molecular Mechanisms Associated with Recurrence

The reactivation of dormant cells represents one of the great tragedies and challenges in BC. Predictive modeling of molecular events during the life of a patient that drive the reawakening of quiescent BC cells in the BM over extended periods is less well-developed than the predictive models based on patient and tumor characteristics at the time of diagnosis outlined above [190]. This section outlines some of the biological events that potentially disrupt dormancy, which have been associated with greater than expected rates of recurrence. The available data are often generated in a variety of systems and cell types, but they support some mechanisms relevant to BC cells in the BM. However, these models cannot predict with certainty when a dormant DTC will awaken, since the mechanisms are likely multifactorial and have significant overlap, making recurrence events appear stochastic [4,191]. 

At the core of the reawakening of dormant cells lies the consensus definition of dormancy. It stipulates that dormant cells must have the capacity to re-enter proliferative states upon withdrawal of dormancy-initiating or dormancy-maintaining factors or upon stimulation by factors that actively drive cells from dormancy to proliferation [106]. Scenario-specific mechanisms of escaping dormancy are rarely clear in patients. Specific events that initiate or permit the initiation of cycle activation signaling can only be presumed from these associations. While triple-negative BCs have a higher tendency to recur in the first 5 years after diagnosis, suggesting a cancer cell-dependent process, hormone-sensitive cancers typically continue to recur stochastically for more than 20 years, suggesting that microenvironmental factors responsible for maintaining dormancy have a predominant role in the process [17]. 

Gene expression comparisons of dormant and growing micrometastases have demonstrated significant increases in the expression of genes involved in the induction and maintenance of dormancy and survival compared to the upregulation of genes involved in proliferation in the awakened cells [192]. Gene expression patterns in primary tumors have demonstrated some propensity for the development of metastatic growth. For example, lncRNA NR2F1-antisense (AS)1 expression in primary tumors was associated with metastatic tumor recurrence [193]. The expression of MSK1 prevents metastatic progression of ER^+^ breast cancer to the bone marrow and may be a potential marker for stratifying patients into a good prognostic group [134]. Direct comparisons of primary tumor and metastatic tumor gene expression profiles have been conducted, but they have been carried out mostly with metastases from other sites than bone. In an analysis of newly diagnosed metastatic breast cancer, gene expression profiles of primary tumors and paired metastases, mostly from the liver, showed that metastases were enriched in estrogen receptor 1 (α) (*ESR1*), *PTEN,* cadherin-1 (*CDH1*), phosphatidylinositol-4,5-bisphosphate 3-kinase catalytic subunit alpha (*PIK3CA*), retinoblastoma-associated protein *(RB1*) mutations, MDM4 regulator of p53 (*MDM4*), MYC proto-oncogene, bHLH transcription factor (*MYC*) amplifications and AT-rich interactive domain-containing protein (*ARID*)*1A* deletions [194]. Metastases exhibit increased clonalities for the driver genes Erb-B2 receptor tyrosine kinase 2 (*ERBB2*) and *RB1*. Subtype switching was also observed in over one-third of the cases, with luminal A/B to Her-2-enriched switching being associated with *TP53* and *PIK3CA* mutations. Actionable targets were present in over half of the patients. Metastases also had lower immune scores and increased immune permissive cells [194]. These data suggest that the comparison of metastases to primary tumors may give way to potential avenues for therapy.

Dormant micrometastases express mesenchymal programs, and when they are reawakened, they undergo MET and begin to proliferate [121]. These cells can serve as launching pads for colonization and re-metastasis [167]. Dormant micrometastases that reawaken and undergo MET change their response to FGF-2 by proliferating [121], in contrast to breast cancer cells expressing an epithelial program [98,121]. Once dormant cells begin to proliferate, they can no longer be redirected into dormancy by FGF-2 [121]. Proliferative signaling that can awaken dormant breast cancer cells may be activated through growth factors including EGF, TGFβ1, integrins, adhesion molecules and stromal remodeling [46]. Nuclear noncoding RNAs called ESR1 locus enhancing and activating noncoding RNAs (ELEANORS), which act as chromatin regulators that upregulate CD44, can induce reawakening and are an independent recurrence risk factor in hormone receptor-positive BC [195].

Among the many variables that have been investigated, factors associated with age- and menopause-related inflammation, oxidative stress, estrogen deprivation-induced elevated cytokines [121,196], activated chemokines [197], activated macrophages [198] and a shift to adipocyte differentiation [199] have been shown to play roles in recurrence. Other potential causes of recurrence include epigenetic reprogramming by the stroma [200], autophagy [201], increased stress hormones and glucocorticoid receptor expression in metastases [202], transient angiogenic bursts [203], systemic response to surgery [204], use of opioids [205], sympathetic signaling [206] and depression through serotonin-induced RUNX2/PTHrP/RANKL signaling [207], among others. The literature cited here describes some of the data that report on these effects. There is a mechanistic overlap among the categories, regardless of how they are organized, as multiple basic processes play roles in each mechanism associated with recurrence.

#### 3.2.1. Inflammation

Multiple factors can induce global inflammatory responses that may be reflected by the eventual reactivation of dormant cells, as outlined in Table 2. The microenvironment-based suppression of cancer cell dysfunction, or parabiosis, mediated by soluble, structural and cellular interactions with cancer cells, including receptor-mediated signaling, intercellular trafficking of exosomes, ions and metabolites via gap junctions, is interrupted by aging and chronic inflammation or injury [208]. Some of these factors are addressed in this study.

A recent review by Manajili et al., 2022 [209], outlines the roles of different categories of immune responses on dormancy initiation and relapse. It delineates the effects on Type I inflammation, consisting of cytokines TNF-α, IFN-γ, IL-17, IL-6/soluble IL-6 receptor α (sIL-6 Rα) and cell-mediated immune response by CD4^+^ Th1 T cells, M1 macrophages and group 1 innate lymphoid cells (ILC-1) to induce dormancy apoptosis or inhibit the proliferation of malignant cells [209]. Type II inflammation, mediated by IL-11, IL-22, IL-33, IL-6/membrane IL-6 receptor α (mIL-6 Rα) and cell-mediated immune response through CD4^+^ Th2 cells, which produce IL-12, M2 macrophages, and group 2 innate lymphoid cells (ILC-2), induces classic anti-inflammatory signaling and tumor relapse [209]. Both type I and type II immune modulations participate in dormancy, if signaling through mIL-6 Rα supersedes signaling via sIL-6 Rα, or if signaling via Th17 or ILC-3 cells predominates in their tumor inhibitory signaling with their interaction with other immune-modulating cells [209]. Both Th17 and ILC-3 cells produce IL-17, which inhibits tumor cell proliferation, and IL-22, which promotes tumor growth and relapse, setting up a balance between growth promotion and inhibition modulated by environmental effects [209]. 

Dormant cells can undergo programmed cell death during their protracted dormancy and attrition. Apoptotic cells promote neutrophil accumulation and the formation of neutrophil extracellular traps (NETs) in a pannexin 1 (Panx1) channel-dependent manner through the release of spermidine, contributing to cancer cell immune escape mechanisms [210]. Inflammation from a variety of sources can induce neutrophils to generate reactive oxygen species and NET formation during the physiological process of fighting infections [211]. NET formation can be induced by sustained lung inflammation from tobacco smoke exposure or nasal instillation of lipopolysaccharide, which is required for the awakening of dormant lung metastases through neutrophil elastase [212]. NET formation also requires MMP9 and cleaved laminin, which induce the proliferation of dormant cancer cells by activating integrin α3β1 signaling [212]. Activated tumor-associated fibroblasts, including BM fibroblasts, induce NET formation through amyloid-β-induced reactive oxygen species signaling pathways and cause tumor cell activation [213].

Although these investigations do not specifically address the role of NETs in activating dormant breast cancer cells in the BM, the mechanisms are consistent with events in the BM that are associated with inflammation or dormant cell death through their prolonged period of attrition [191] or catecholamine-inducing events. For example, the expression of ADRB3, which mediates catecholamine-induced activation of adenylate cyclase through G proteins, is three times more frequent in breast cancer tissues than in adjacent noncancerous tissues and correlates with the TNM stage and poor prognosis [214]. ADRB3 expression is elevated in disseminated cycle-activated cancer cells, lymphocytes, inflammation-suppressing myeloid-derived suppressor cells (MDSCs) and NETs in patients [214]. Hence, ADRB3 promotes the expansion of MDSC through BM mobilization and inhibition of immature myeloid cell differentiation and promotes the expansion of ER^+^ human breast cancer cells [214]. Initial reactivation or further seeding can activate NET formation by attracting neutrophils and MDSC infiltration and secretion of neutrophil elastase, hijacking the normal host-protective immune system and participating in the feed-forward promotion of inflammation in the stromal microenvironment [215].

Activated macrophages can induce dormancy reactivation as well [198]. NETs [216], macrophages [217] and EGF signaling [218] induce the upregulation of VCAM-1, which is partially dependent on NF-κB signaling and can promote the transition from indolent micrometastases to overt metastases [219]. NETS can induce the proinflammatory adhesion molecules VCAM-1, intercellular adhesion molecule (ICAM)-1, E-selectin, IL-1β, IL-6 and chemokine CXCL1 [216]. Macrophages significantly upregulate the expression of VCAM-1 and elicit a proinflammatory response through NF-κB, TNFα, IL-1β and IL-6 [217]. EGF treatment upregulates VCAM-1 and enhances the interaction between macrophages and cancer cells [218]. Aberrant expression of VCAM-1 in dormant micrometastases recruits monocytic osteoclast progenitors through the binding of integrin α4β1 and increases local osteoclast activity, initiating the cycle of bone destruction and micrometastasis reawakening [219]. 

Inflammation associated with infectious processes may generate an environment that stimulates the reawakening of dormant cancer cells as well. Severe acute respiratory syndrome resulting from coronavirus 2 (SARS-CoV-2) can activate neutrophils and monocytes/macrophages, activate NETs and induce lymphopenia and uncontrolled production of pro-inflammatory cytokines [220]. NETs released by activated neutrophils may be involved in DTC reawakening in COVID-19 patients [220]. In another venue and model, the stromal immune response through secretion of IFNγ can activate stromal fibroblasts, as in the case of hepatic stellate cells, to block fibroblast-secreted CXCL12-mediated induction of NK cell suppression of metastatic outgrowth [221].

Additional proliferative signaling may induce reawakening through the induction of periostin in metastatic niche fibroblasts by tumor cells, which in turn activates tumor-initiating cells [222] and remodels the ECM with aging and inflammation [42,129]. This effect may generate a tumor-promoting collagen I-enriched fibrotic environment, which signals through integrin β1 activation of Src and FAK [223].

**Table 2:** Proliferative and inflammatory mechanisms of dormant breast cancer cell reawakening.

	Mechanism	Vehicle and Function	Signaling	References
Proliferative signaling	Dormant cell cycle activation	EGF, TGFβ1, integrins, adhesion molecules, periostin, stromal remodeling collagen I fibrotic niche ELEANORS	proliferative signaling chromatin regulation, upregulated CD44	[42,46,121,129,222] [223] [195]
Inflammation	Type I inflammation	TNF-α, IFN-γ, IL-17, IL-6/sIL 6Rα CD4^+^ Th1 T-cells, M1-macrophages, ILC-1	Dormancy if mIL-6 Rα signaling > sIL-6Rα signaling Dormancy if tumor inhibitory Th17 or ILC-3 cell signaling predominates IL-17 IL-22 promotes relapse	[209]
	Type II anti-inflammatory classic signaling	IL-11, IL-22, IL-33, IL-6/mIL-6 Rα, CD4^+^ Th2 cells, IL-12, M2-macrophages, ILC-2	Dormancy if mIL-6 Rα > sIL-6 Rα signaling Dormancy if tumor inhibitory Th17 or ILC-3 cell signaling predominates IL-17 IL-22 promotes relapse	[209]
	NETS	Panx1 ADRB3 VCAM-1, ICAM-1, E-selectin, IL-1β, IL-6, CXCL1	Spermidine, immune escape, MMP9, cleaved laminin, activated integrins ROS Expanded MDSCs, cycle activates BC cells feed-forward inflammation α4β1-induced osteoclast activity	[210,212,213,214,215,216,219]
	Macrophages	VCAM-1	NF-κB, TNFα, IL-1β, and IL-6 α4β1-induced osteoclast activity	[198,217,219]
	EGF	VCAM-1	NF-κB α4β1-induced osteoclast activity	[218,219]
	COVID-19 infection	NETs, monocytes/macrophages	pro-inflammatory cytokines	[220]
	Stromal immune response	INFγ	activated stromal fibroblasts, blocked CXCL-12-NK cancer cells suppression	[221]

#### 3.2.2. Aging

Aging and post-menopausal loss of estrogen [224,225] induce a shift to adipose differentiation [199], increased BM adipocyte content [226], increased expression of RANKL [227] and a decreased number of bone-forming osteoblasts [228], all of which contribute to dormant breast cancer cell reawakening, as outlined in Table 3. This shift is due to a switch in the differentiation potential of MSCs from an osteogenic to adipogenic preference with aging [59] involving TGFβ/BMP signaling and the transcription factor PPARγ2 [228]. Osteoprogenitor cells have decreased proliferative capacity with aging, suggesting that decreased osteoblastic cell number, not function, leads to an age-related decrease in bone formation [229]. Aging induces senescence in MSCs through the loss of expression of FOXP1 [230]. Homeobox (HOX)B7, which is a master regulator highly expressed in youth, drives MSC behavior over the mammalian lifespan, including promoting proliferation, FGF-2 synthesis and osteogenic differentiation, but declines progressively in aging bone marrow MSCs [231]. HOXB expression in MSCs is inhibited by progressive upregulation of miR-196 levels with aging and results in diminished MSC proliferation, senescence induction, osteogenesis inhibition and a dramatic reduction in FGF-2 expression [231].

Older normal human fibroblasts have elevated levels of arachidonic acid 12-lipoxygenase (ALOX12) and its mitogenic metabolite, 12-(S)-hydroxy-5,8,10,14-eicosatetraenoic acid (12-(S)-HETE), compared to their younger counterparts [232]. Consequently, older fibroblasts preferentially induce MAPK signaling and increase cellular metabolism, lower oxidation rates and enhance proliferation and resistance to radiation in tumor cells in co-culture [232]. 

The role of aging in the capacity of the BM microenvironment to support dormancy or promote the reawakening of BC micrometastases can be modeled in 18-month-old mice [233]. N-cadherin (Cadh2) expression changes in osteoblasts with aging [233] and may decrease pro-osteogenic Wnt5a and Wnt10b expression in MSCs, steering them to adipogenesis [234,235,236,237]. These events promote inflammation [121,196,212] and the secretion of soluble factors that stimulate the production of inflammatory cytokines. These include IL-6 and IL-8 [121], Il-1b, Il-6, Il-27, Il-1f9, C-C motif chemokine ligand (CCl)4 and Ccl5, TNF superfamily member 14 (Tnfsf14), lymphotoxin β (Ltb) [97] and TGF-β1 signaling by BM mesenchymal cells [121,159]. These, in turn, can reactivate dormant DTC proliferation, renew CD133+ cancer cells and endocrine resistance by IL-6/Notch signaling [238] through the IL-6 receptor gp130/gp80 and activated STAT3 [239,240], through VEGF [241] via PI3K/Akt signaling [242], SMAD2 and 3 [121], and the EGFR and ERK pathways [243].

The bone marrow environment of mice aged 55–65 weeks permits expansion of transplanted human ER^+^, triple-negative and Her2-amplified BC cells, and retains non-cycling quiescent cells to a far lesser extent than that of young 4–6 week old mouse recipients [97]. Injection of aged mouse bone secretomes into mice with transplanted human breast cancer cells induces the rapid expansion of the transplanted breast cancer cells in bones, without demonstrating a difference in the number of quiescent cells compared to mice stimulated with young bone secretomes. Factors involved in the maintenance of stem and cancer cell quiescence, such as Bmp4, Bmp6, Bmp7, Kit ligand, TGF-β2, Dkk-1, Dkk3 and thombospondin2 (Thbs2), are higher in the bones of young mice than in old mice, but radiation markedly upregulates them in old mice [97]. The stem cell maintenance factors are synthesized by pericytes, which markedly decline with age. Radiation and cytotoxic chemotherapy cause an increase in bone marrow pericytes mediated through CD31^hi^/endomucin (Emcn)^hi^ type H endothelial cell secretion of platelet-derived growth factor (PDGF)-BB, which binds to pericyte PDGFRβ, promotes their proliferation and results in resistance to therapy. These data illustrate some of the mechanisms responsible for the progressive loss of support for the dormancy of breast cancer stem cells in the bone marrow with age [97].

A murine melanoma metastasis model points to the inhibition of non-canonical Wnt5a signaling, which has potential relevance in the reawakening of dormant breast cancer cells in the BM [244]. Wnt5a is a promoter of prostate cancer dormancy in the BM through a mechanism by which it represses canonical Wnt signaling that promotes tumor progression [142]. In this model, aged skin fibroblasts release secreted frizzled-related protein (sFRP)2, which triggers Wnt5a expression in tumor cells and promotes dormancy signaling via the receptor tyrosine kinase AXL, which in turn limits MERTK signaling and drives tumor dormancy [244]. sFRP2 is an antagonist of canonical Wnt signaling and leads to decreased β-catenin and the loss of redox effector apurinic/apyrimidinic endonuclease-1/redox factor (APE)-1, which may attenuate the response to DNA damage induced by reactive oxygen species and induces resistance to targeted therapy in dormant cells in another model of melanoma [245]. In the lung, however, metastatic cells are reactivated by aged fibroblasts through secreted sFRP1, which is an antagonist of Wnt5a, resulting in decreased AXL signaling, enhanced MER activation and tumor cell proliferation [244]. Wnt5a and Wnt10b have pro-osteogenic roles in the BM, and their expression is decreased in aging osteoblasts and MSCs, promoting adipogenesis and tilting the balance in the direction of dormant cell reawakening [234,235,236,237].

#### 3.2.3. Loss of FGF in Stroma

We and others have demonstrated significant roles for FGF-2 generated by bone marrow stromal MSCs in maintaining BC and other solid tumor stem cell phenotypes and dormancy [98,113,120,121,130], and in maintaining MSC capacity for osteogenic differentiation [116,117,118]. FGF-2 export and deposition in the endosteal niche appear to be one of the main contributors to the maintenance of dormancy in ER^+^ breast cancer cells, and the loss of this function in aging MSCs contributes significantly to the observed stochastic recurrence of bone marrow micrometastases. Overall, aging induces a decrease in the quantity and quality of bone marrow MSCs, reducing their capacity for damage repair, and endows them with reduced proliferation and paracrine signaling, including the secretion of EGF, FGF-2, HGF and IGF, and a state of increased oxidative stress [246]. These effects depend on long non-coding RNA-p21 via the suppression of β-catenin [246]. FGF-2 mRNA levels are decreased by more than 50% in aged MSC compared to young MSCs [246]. In fact, both FGF-1 and FGF-2 inhibit adipogenic differentiation and induce the expression of MMP-13, diminish tissue inhibitor of metalloproteinases (TIMP)1 and modulate collagen turnover by human BM MSCs in collagen gels [247]. Interestingly, FGF-1 and -2 also inhibit osteogenic differentiation in this model [247]. MMP-2-mediated fibrillary fibronectin degradation is also necessary for escaping dormancy [103]. 

In addition to switching their osteogenic differentiation potential in the direction of adipocytic differentiation and ceasing to secrete factors directly supporting dormancy, such as FGF-2, aged MSCs also lose the ability to provide regenerative effects to other organs [231,246,247,248,249,250]. The expression of FGF-2 in MSC is necessary for its role in osteogenic differentiation [251]. Genetic knockout of FGF-2 results in an adipogenic BM mediated by the induction of PPARγ2 and the downstream target genes apetala (aP)2 and adiponectin, whereas incubation with FGF-2 restores their osteogenic differentiation potential [251]. Of note, the regenerative effects of aged MSCs can also be restored with in vitro incubation with FGF-2 [252,253] or with FGF-2 combined with retinoic acid and sonic hedgehog (SHH) [254].

#### 3.2.4. Increased Adipogenesis

The interaction of adipocytes with metastatic breast cancer cells in the bone marrow and the establishment of a cancer-hospitable microenvironment have been expertly reviewed by Liu et al., 2020 [255]. While the comprehensive effect of the microenvironment is to suppress cancer cells that arrive in the bone marrow and induce mesenchymal and stem-like dormant phenotypes in the surviving micrometastases in the stem cell niches, as outlined above, adipocytes appear to fuel tumor-promoting effects. Two populations of marrow adipose tissue exist in the BM: the constitutive adipocytes that begin to form before birth and mature during early life, and the regulated adipose tissue that develops later and expands with age in areas of active hematopoiesis [256]. The regulated adipose tissue and its functional support of surrounding cells expand with age, systemic disease, anorexia, obesity, osteoporosis, hyperlipidemia, estrogen deficiency and treatment with glucocorticoids and thiazolidinediones [257,258,259]. Prolonged administration of glucocorticoids shifts the differentiation of MSCs to adipocytes and may play a pathological role in bone marrow adipogenesis [260]. In addition, the loss of beta-catenin expression in preosteoblasts, which is necessary for osteoblast differentiation, may lead to a shift in cell fate from osteoblasts to adipocytes in aging bone marrow [261]. With aging, the bone marrow acquires an increased adipocyte content at a rate of about 7% per decade [262], with men achieving 50% fat content in their 40s and women in their early 60s [263,264] due to altered differentiation programs of MSCs [59,199,228]. MSCs increase the expression of glycophorin, syntaxin-3, PPARγ and CCAAT enhancer binding protein alpha (C/EBP-α) to shift their differentiation program to adipogenesis [59]. Most breast cancer recurrences take place at a later age, as the ratio of adipocytes to hematopoietic cells in the bone marrow continues to increase with age [263,264].

Adipocytes stimulate breast cancer cells by secreting cytokines that bind to their corresponding receptors on breast cancer cells. These include secreted leptin, which binds to receptor Ob-R, stimulates JAK/STAT3 and PI3K/Akt signaling and also activates ER and Her2 receptors independently of their ligands [255]. The secreted adiponectin binds to the Adipo-R receptor to activate PI3K/Akt and MAPK/ERK. TNF-α binds to TNFR in breast cancer cells and activates MAPK/ERK and NF-kB, and IL-1β secreted by adipocytes binds to IL-1R and upregulates and activates NF-kB and CREB via its receptor IL-1R [255]. Coactivation of the ER and the canonical NF-κB pathway promotes the features of aggressive ER^+^ breast cancer [156]. IL-6 binds to IL-6R, resistin binds to its toll-like receptor (TLR)-4 and adenylate cyclase-associated protein (CAP)1, and both stimulate JAK/SATA3 signaling [255]. Fatty acid-binding protein (FABP)4 is internalized by BC cells and enhances JAK/SATA3, PI3K/Akt, and MAPK/ERK signaling, visfatin stimulates MAPK/ERK and Notch signaling, and chemerin upregulates RhoA/ROCK activation via its chemokine-like receptor (CMKLR)1 [255]. RhoA activation is significant, as its inactivation plays a major role in maintaining the adherent, non-motile phenotype of dormant cells with mesenchymal programs [120,121]. The rest of the signaling by these adipokines activates proliferation, EMT, stemness and angiogenesis, and plays a role in tipping the balance from dormancy to reactivation in micrometastases [255]. The upregulation of the fatty acid transporter CD36 [265,266] or its activation through cysteine oxidation [267] increases the uptake and accumulation of β-oxidized lipids and promotes the reawakening of dormant cancer cells [265,266]. In addition, oxidative stress induces P450 epoxygenases to synthesize epoxyeicosatrienoic acids and causes cells to exit dormancy [268]. The roles of the modulations in the oxidative state of the metastatic microenvironment are expertly reviewed by Qin et al., 2022 [140].

Mature adipocytes also release extracellular vesicles that promote breast cancer cell malignancy by enhancing growth, motility, invasion, EMT traits and stem cell-like properties in both ER^+^ and triple-negative BC in vitro, and aid breast tumor cells in lung metastatic colonization in vivo in tail vein-injected cells in a murine model in a hypoxia-inducible factor (HIF)-1α-dependent manner [269]. The effects depend on the extracellular vesicles and only occur with vesicles derived from obese adipocytes, whereas undifferentiated adipocytes fail to induce tumor aggressiveness and HIF-1α expression [269]. 

In a clinical correlate, the recurrence risk for breast cancer increases by 2% for every 1 kg/m^2^ increment in body mass index, as derived in a meta-analysis of 21 clinical studies [270]. An epidemiologic review of preclinical and clinical investigations on the roles of obesity in recurrence and death from breast cancer highlights clinical investigations on serum levels of leptin as a potential biomarker for primary or secondary outcome measures in breast cancer patients after behavioral dietary and exercise interventions [271]. Indeed, exercise prevents cancer recurrence, and under limited glucose conditions, active stroma consumes significantly more glucose at the expense of the tumor [272]. A 20-year prospective study of a large cohort of initially cancer-free participants revealed that physical exercise prior to cancer initiation significantly reduced the likelihood of highly metastatic cancer. Exercise induces the metabolic reprogramming of internal organs, increases nutrient demand and protects against metastatic colonization by limiting nutrient availability to the tumor, generating exercise-induced catabolic processes, glucose uptake, mitochondrial activity and glucose transporter (GLUT) expression, creating a metabolic shield against metastasis [272].

#### 3.2.5. Estrogen Deprivation

Estrogen depletion paradoxically awakens ER^+^ breast cancer cells and promotes their proliferation in a BM endothelial cell dormancy model [273]. Estrogen depletion induces BM niche cells to produce angiopoietin-2, which destabilizes the niche by interfering with angiopoietin-1/Tie2 signaling and promotes ER^+^ tumor cell survival via integrin β1 [273]. The association between angiopoietin-2 expression in human breast cancer samples and distant metastases only occurs in patients who undergo adjuvant estrogen depletion therapy [273]. Estrogen deprivation in cultured BM stromal monolayers induces the secretion of IL-6 and IL-8, activates TGFβ and TNFα signaling and promotes the growth of breast cancer colonies [121]. These inflammatory cytokines induce dormant metastasis reawakening, stem cell renewal and endocrine resistance [238]. In our in vitro model of ER^+^ breast cancer dormancy, FGF-2 induces a complete loss of ER expression in dormant cells, which does not recover upon reawakening [121].

**Table 3:** Effects of aging on dormant breast cancer cell reawakening in the bone marrow.

	Mechanism	Vehicle and Function	Signaling	References
Aging	Estrogen deprivation	MSCs	angiopoietin-2, disrupted angiopoietin-1/Tie2 signaling, ER^+^ tumor cell survival via integrin β1 secreted IL-6, IL-8, activated TGFβ, TNFα signaling	[224,225,273] [121]
	Shift to adipose differentiation	increased BM adipocytes, RANKL, decreased bone forming osteoblasts - adipocyte leptin - increased β-oxidized lipid uptake - oxidative stress - adipocyte extracellular vesicles	- switch in MSCs differentiation potential from osteogenic to adipogenic, - TGFβ/BMP, PPARγ2 signaling, - Ob-R, FABP4, JAK/STAT3, PI3K/Akt, ERK, Rho/ROCK, Notch, TNF-α, ERK, NF-kB, IL-1β, CREB, IL-6, resistin - ligand-independent ER and Her2 receptor activation - CD36 cysteine oxidation - P450 epoxygenase- induced epoxyeicosatrienoic acid synthesis - Promotes ER^+^ and ER^−^ BC cell proliferation, motility and metastasis - Hif1α	[59,199,226,227,228,255,263,264,265,266,267,268,269]
	Decreased osteoprogenitor cell proliferative capacity			[229]
	MSC senescence	loss FOXP1 expression, HOXB7 declines	miR-196 upregulation	[230,231]
	Increased fibroblast metabolism, lower oxidation	Increased ALOX12	Increased ERK signaling, radiation resistance	[232]
	Niche fibroblasts	Increased periostin		[222]
	Increased MSC N-cadherin	MSCs steers to adipogenic differentiation	decrease in pro-osteogenic Wnt5a and Wnt10b signaling - decreased AXL dormancy signaling, - enhanced MERTK tumor promoting signaling. - Altered balance of sFRP2 canonical protumorigenic Wnt/β-catenin antagonist and sFRP1 dormancy sustainer	[142,233,234,235,236,244,245]
	inflammation	MSCs	IL-6, IL-8, Il-1b, Il-6, Il-27, Il-1f9, CCl4, Ccl5, Tnfsf14, Ltb, TGF-β1 signaling, SMAD2 and 3, - CD133^+^ cancer cell renewal - IL-6/Notch induced endocrine resistance through gp130/gp80, STAT3, VEGF, PI3K/Akt signaling, EGFR and ERK	[97,121,159,196,212,238,239,240,241,242,243]
	Loss of stem cell maintenance	Pericytes, decline with aging	Decrease in Bmp4, Bmp6, Bmp7, Kit ligand, TGF-β2, Dkk-1, Dkk3, Thbs2	[97]
	FGF-2 synthetic loss by MSCs	- Lose damage repair capacity, proliferation, EGF, FGF-2, HGF, IGF signaling, FGF-2 expression, - increase oxidative stress - lose FGF-1- and FGF-2-mediated inhibition of adipogenesis	- MSC senescence through lnRNA-p2 β-catenin signaling suppression - decreased MMP-13, TIMP1, MMP-2-mediated fibrillary fibronectin degradation - induced collagen turnover - PPARγ2 adipogenic signaling	[103,231,248,249,250]

### 3.3. Stromal Injury 

The effects of stromal injury on dormant BC cell reawakening are outlined in Table 4. Chronic exposure to toxins from petrochemicals can induce elevated levels of IL-8 and decrease DNA repair capacity [274]. Diesel exhaust particles increase inflammatory cytokine production and decrease functional chemotaxis in M1 and M2 macrophages in exposed individuals [275]. Chemotherapy, biological agent therapy and radiation can induce injury and secretory senescence in stromal fibroblasts, including those in the BM [121,276,277,278,279]. The induction of DNA damage response induces NF-κB activation through nuclear translocation or cytosolic TRAF-interacting protein with forkhead-associated domain (TIFA), its accumulation on damaged chromatin and the resulting secretion of classic NF-κB targets, including IL-6 and IL-8 [277]. Toxic agent-induced secretory senescence-induced ATM and NF-κB activation and IL-6 and IL-8 secretion can also be induced by chromatin remodeling rather than physical breaks in DNA, which can result in osteopontin activation via HDAC inhibitor treatment [280]. Secretory senescence can also result from an environment generated by dietary fat and IL-1 [281]. Secretory senescence can induce tumor progression and potential reawakening of micrometastases [121,282]. Secretory senescence in osteoblasts with the associated secretion of IL-6 induces colonization of disseminated BC cells and osteoclastogenesis [283].

Stromal co-cultivation models can use a genetic model of induced senescence by the expression of p27^Kip1^, which is sufficient to recapitulate the senescent phenotype in human cells [284] and induce cancer cell reawakening [283]. In fact, a virtually unlimited number of variable interventions can be introduced in a 2D stromal co-culture to test hypotheses on the reactivation of cancer cells [37]. Stimulation of dormant cells with inflammatory cytokines after being released from FGF-2 stimulation results in the reactivation of dormant cells into growing cells with spindle-like morphology and downregulation of the mesenchymal gene expression pattern in an in vitro model [121]. 

Cancer cells or other co-cultured cells in the stroma can be stained in situ with fluorescence-linked antibodies, phallacidin, carboxyfluorescein succinimidyl ester (CFSE), or detached for flow cytometric analysis, or other techniques to determine signaling, co-localization, actin rearrangement and quiescence [37]. Colony regrowth can be determined by manual counting, and colony size can be determined using photography and the Fiji Cell Profiler software ImageJ Win64.

We investigated the role of stromal injury and inflammation in our in vitro model of dormancy. Our studies demonstrate that stromal injury can cause the release of IL-6 and IL-8, the activation of TGF-β1 and promote the growth of dormant ER^+^ BC cells [121]. Exogenous IL-6, IL-8 and TGF-β1 also reactivate dormant BC colonies in the model described above when FGF-2 is removed after 8 days in culture, with the reactivated cells taking on a mesenchymal appearance. However, prior treatment of cells with FGF-2 already activated a mesenchymal gene expression pattern and behavior, characterized by the downregulation of E-cadherin, upregulation of N-cadherin and SLUG and downregulation of ER, yet they continued to retain their dormant appearance when FGF-2 was removed before reawakening. Cytokines and TGF-β1, which reawakened the dormant clones, partly diminished the mesenchymal gene expression profile of the cells and completely eliminated ER expression [121]. Oxidative (H_2_O_2_)-, hypoxic (carbonyl-cyanide m-chlorophenylhydrazone (CCCP))- and estrogen deprivation with fulvestrant (ICI182780)-induced injury to murine BM stroma resulted in the enhanced outgrowth of co-cultivated BC colonies and activation of TGF-β and TNF-α signaling pathways in murine BM stromal cells. Oxidation and estrogen deprivation, but not hypoxia, also induced IL-6 secretion in the stroma [121].

Oxidative injury to the stroma results in the secretion of inflammatory cytokines and cell cycle activation of dormant cancer cells [121]. Oxidative stress can awaken dormant tumor cells by activating lipid transport receptors to enhance lipid metabolism. The transition of the microenvironmental redox status from hypoxia to inflammation is also an essential awakening mechanism [140]. Co-culture of MCF-7 cells on murine stroma induced the secretion of IL-6 and IL-8 by stromal cells exclusively [121], demonstrating a feed-forward stimulation mechanism for cancer cell growth once reactivation is initiated. Once dormant cells begin to proliferate, they can no longer be induced back to dormancy by FGF-2 in this model [121].

Mesenchymal to epithelial conversion in vivo reinitiates proliferation, potentially mediated by heterotypic adherence junctions between newly re-expressed tumor-cell E-cadherin (CDH1) and osteoblast N-cadherin (CDH2) [285]. As BC cells begin to proliferate, they in turn induce stroma to secrete interleukins, TNF-α, [121] and other factors, such as secretomes, nutrients, metabolites and inflammatory cells [286], creating a feed-forward loop to promote recurrence. 

### 3.4. Hypercoagulable State

The association between occult cancer and the predisposition to venous thrombosis has been recognized for decades, and a causal effect of injected thrombin on reawakening cancer cells in a pulmonary metastasis animal model has been demonstrated [287]. The effects are outlined in Table 4. Thrombin has a protease-activated receptor (PAR-1) binding site on tumor cells [288], activating them to bind more tightly to fibronectin, platelets, vWF and endothelial cells, and endowing them with a greater metastatic potential to the lungs in experimental models [289,290]. Thrombin stimulates cancer cell proliferation by downregulating p27^KIP1^ and inducing S-phase kinase-associated protein (Skp)2, cyclins D and A and microRNA-222, which inhibits p27^KIP1^ [291]. In addition, thrombin can act as a mitogen for fibroblasts, endothelial cells and smooth muscle cells [292,293], and small concentrations can induce angiogenesis through VEGF and its receptor [294]. Indeed, the specter that thrombin is a tumor-promoting agent and that endogenous anticoagulants such as antithrombin III, protein C, α2-macroglobulin, thrombomodulin and others may act to maintain dormancy in cancer cells that may have been transformed years earlier but have not become clinically detectable has been suggested from clinical evidence on anticoagulation, hypercoagulability and cancer incidence studies [294]. 

The endogenous thrombin potential, which is the net amount of thrombin that the plasma can generate, and the thrombin generation peak are significantly higher in newly diagnosed, resected high-risk breast cancer patients than in normal controls [295,296]. The thrombin potential provides strong contributions to identifying patients at a high risk of early disease recurrence compared to patients with late or no recurrence [295,296]. Early thrombin generation potential was an independent risk factor along with mastectomy, luminal B Her2^−^ and triple-negative subtypes via multivariable analysis [296].

Furthermore, elements of a hypercoagulable state, as determined by coagulation factor VIII (FVIII) and D-dimer (DD) levels, along with the classic staging factors of age and pathologic tumor size at the time of diagnosis, prior to any therapy or surgery in stage I-II breast cancer patients, are predictive of overall survival [297]. The FVIII levels, along with staging criteria of lymph node involvement and ER expression, also affect disease-free survival [297].

Viral infections have been associated with hypercoagulable states and thrombotic effects. These include SARS-COVID-19, which leads to endothelial damage, microvascular thrombi, elevated D-dimers and platelet hyperactivation [298,299,300,301], as well as the systemic inflammation noted above [220,302]. Hypercoagulable syndromes due to endothelial activation have been associated with other viral infections, including human herpes virus-6 (HHV-6), Epstein–Barr Virus (EBV) and cytomegalovirus (CMV) [303,304]. Other prothrombotic viruses that infect endothelial cells and induce vWF activation and microangiopathy include a variety of strains of influenza viruses A and B, parainfluenza-1, respiratory syncytial virus (RSV), parvovirus B19, HIV and hepatitis B virus [305]. Other viruses that infect platelets and can cause thrombotic events include Marburg, Ebola, Crimean Congo, Hantavirus, Yellow Fever and Lassa fever [305]. Many of these viruses activate multiple elements of the coagulation cascade and generate a hypercoagulable state, resulting in microthrombi and thromboses in various organs [305]. Various studies have suggested the use of chronic anticoagulants in certain COVID-19 infections [299,302], but the use of antithrombotic therapy for preventing the recurrence of BC after viral infections has not been investigated.

**Table 4:** Effects of stromal injury and a hypercoagulable state on dormant BC cell reawakening.

	Mechanism	Vehicle and Function	Signaling	References
Stromal injury				
	Petrochemicals		Elevated IL-8, decreased DNA repair	[274]
	Diesel exhaust		- increased inflammatory cytokines - decreased M1 and M2 macrophage chemotaxis	[275]
	Chemo-, bio- and radiation-therapy	Stromal fibroblasts	injury and secretory senescence	[121,276,277,278,279]
	- HDAC inhibitor	- secretory senescence - chromatin remodeling rather than physical breaks in DNA	ATM, NF-κB, IL-6 and IL-8 osteopontin activation	[280]
	DNA damage response		NF-κB activation, TIFA, damaged chromatin, NF-κB, IL-6, IL-8	[277]
	Dietary fat, IL-1	Secretory senescence		[281]
	Osteoblast senescence	P27^Kip1^ secretory senescence	IL-6, osteoclastogenesis	[283,284]
	Oxidative and hypoxic stress		- TGF-β, TNF-α, IL-6 - lipid transport receptors, lipid metabolism	[121,140]
	Colonization, feed-forward stromal injury	Secretory senescence	IL-6, IL-8, TNF-α, secretormes, nutrients, metabolites, inflammatory cells	[121,286]
Hypercoagulable state				
	Thrombin	PAR-1 on cancer cells	Enhanced binding to fibronectin, platelets, vWF, endothelial cells	[288,289,290]
			Downregulated, inhibited p27^KIP1^, induced Skp2, cyclins D and A, and miRNA-222	[291]
			Mitogenic to fibroblasts, endothelial cells, and smooth muscle cells	[292,293]
			angiogenesis through VEGF	[294]
		increased thrombin potential, and thrombin generation peak in high-risk BC	High recurrence potential	[295,296]
	FVIII, D-dimer levels	predictive of overall and disease-free survival		[297]

### 3.5. Surgery, Associated Angiogenesis, Inflammation and Catecholamines

One hypothesis poses the possibility that a smoldering inflammation in the metastatic site, induced by any of a number of factors discussed above [306], may initiate angiogenic bursts from fibroblasts or myeloid progenitors in the microenvironment [203]. The spike of angiogenic factors may not be sufficient to induce angiogenesis by itself but may cross the threshold for the induction of angiogenesis factors by tumor cells [203]. The binding of activated endothelial cell tips through TGF β1 and periostin may induce the proliferation of dormant BC cells [159]. Disseminated breast cancer cells, either early after dissemination or after a period of dormancy in the endosteal niche, translocate to the endothelial niche where they spread on the capillaries, displacing resident pericytes [307]. Tumor cells spread through the cell adhesion molecule L1 (L1CAM) and activate the mechanotransduction effectors Yes-associated protein (YAP) and myocardin-related transcription factor (MRTF) by engaging integrin β1 and integrin-linked kinase (ILK), L1CAM and YAP signaling, enabling the outgrowth of metastasis-initiating cells [307].

The normal physiological bone marrow status is one of low oxygen tension [87]. However, episodes of increased hypoxia due to a variety of causes can induce enhanced angiogenesis, perhaps mediated by local macrophages that secrete Hif-1 and -2 and other angiogenic factors that may initiate endothelial sprouting, which can lead to the cell cycle activation of dormant cancer cells [308]. 

A hypothesis was put forward that a subset of relatively early recurrences in breast cancer among younger women is due to the removal of angiogenesis inhibitors from the primary tumor, in conjunction with the generation of angiogenic growth factors and surgery-induced inflammatory responses to surgical wounding [309]. Animal models have demonstrated that surgery does induce angiogenesis through the elimination of angiostatin as a tumor-originating factor in systemic angiogenesis, but it has not been substantiated as a cause of recurrence in humans [310]. A small study demonstrated that mastectomy induces elevations in a number of circulating angiogenic factors, elevated angiogenesis and growth factor gene expression changes in the blood [311]. The hypothesis also proposed that surgery-induced angiogenesis may be a potential partial cause of the increased rates of race-based differences in survival in premenopausal women, where BC surgery rates in AA patients are greater than those in European-descent patients due to the differences in the incidence of breast cancer between the races in that age group [312]. These hypotheses have not been tested fully and remain controversial [313,314]. However, since their publication, additional data have been generated that support a potential role for surgery-induced physiological responses on the status of dormant micrometastases.

#### 3.5.1. Tumors Produce Metastasis-Promoting Factors That Are Eliminated with Tumor Removal

In a mouse model, a minimally invasive procedure to remove human triple-negative cell line-generated xenograft tumors eliminated tumor-secreted factors, including IL-6, IL-8, VEGF, EGF, PDGF-AA, MIF, SerpinE1, M-CSF, focal adhesion, metalloprotease and apoptosis regulation processes [315]. The procedure induced regression of spontaneous micrometastases into a non-growing dormant state, but not that of larger metastases [315]. In addition, in vivo supplementation with tumor secretomes diminished this regression, suggesting that primary tumor-secreted factors promote early metastatic growth [315]. Indeed, neutralization of IL-8, PDGF-AA, Serpin E1 plasminogen activator inhibitor (PAI)-1 and MIF individually antagonized secretome-induced proliferation, and their simultaneous blockade in vivo in the presence of the primary tumor arrested the development of micrometastases [315].

#### 3.5.2. Surgery Induces Catecholamines and Inflammatory Factors That May Promote the Growth of Dormant Micrometastases

In contrast to the minimally invasive tumor removal experiments above, observations that surgical removal of primary tumors gives rise to the outgrowth of metastases in some patients and in multiple animal models have been reported for more than one hundred years [316]. These reports postulate that the causes are two-fold: one that eliminates angiogenesis inhibitors produced by the primary tumor, and the second that is due to surgery-induced production of growth factors, angiogenesis factors, inflammation and catecholamines that promote metastasis outgrowth (Table 5). 

The net result that primary surgery promotes metastasis outgrowth is supported by numerous mathematical models [316], which undertake no a priori assumptions about the biology of the process but simply model the data and are true regardless of the size of the metastases [316]. According to one mathematical model, surgery stimulates the escape from dormancy, promotes angiogenesis and accelerates metastatic growth in a fraction of breast cancer patients [317]. In fact, studies report that anti-inflammatory treatment after surgery can cause delays in the recurrence of dormant metastases, providing support for the surgically-induced inflammatory state as a contributor [318]. In an experimental murine model, the systemic inflammatory response induced by surgery promotes the emergence of tumors whose growth is otherwise restricted by a tumor-specific T-cell response, and perioperative anti-inflammatory treatment markedly reduces tumor outgrowth [204]. Resection of a primary tumor may also eliminate dendritic cells that generate activated cytotoxic T cells responsible for metastatic cell suppression, and their elimination may diminish an element of immune suppression that contributes to dormancy [319]. In addition, the adrenergic and inflammatory effects of surgery may induce temporary immunosuppression through the disruption of T-lymphocytes, macrophages, NK cells and monocyte dysfunction [320].

In the same animal model that demonstrated that primary tumors promote the growth of metastases through the secretion of growth factors and removal of the tumor through a minimally invasive procedure suppresses them, the data also suggested that major surgery accompanying tumor removal generated high levels of perioperative catecholamines (CAs) and prostaglandins (PGs), specifically PGE2, which mediate numerous pro-metastatic effects of stress and surgery [321]. In this scenario, adding a laparotomy procedure to the minimally invasive removal of the primary tumor from the animals caused an increase in IL-6 and IL-8 levels, elevated NF-κB, reduced IRF1 activity in excised tumor transcriptomes and initiated an outbreak of micrometastases [322]. The secretion of growth factors IL-6, IL-8, and VEGF was markedly enhanced by the β-adrenergic agonist epinephrine and metaproterenol and by PGE2, while cortisol reduced their secretion. Perioperative treatment with propranolol, a β-adrenergic inhibitor, and etodolac, a semi-selective cyclooxygenase (COX)-2 inhibitor, blocked the secretion of pro-metastatic factors [322]. COX-2 inhibitors, such as celecoxib, target BC stem cells by inhibiting the synthesis of PGE2 and downregulating the Wnt pathway activity [323]. Since COX-2 inhibitors may have unintended adverse effects on cardiac toxicity, alternative ways for suppressing PGE2 production via the inhibition of miR-155 have been studied [324]. miR-155 has a dual effect: reprogramming PG metabolism by upregulating PGE2-producing enzyme PTGES through the miR-155-cMYC axis and the enzyme PTGES2 through Kruppel-like factor (KLF)4, and by downregulating PGD2-producing enzyme PGD2 Synthase (PTGDS) [324]. The miR155 antagonist MRG-106 underwent a phase-1 clinical trial for safety and may serve as a candidate to modulate PGs in BC recurrence.

In a randomized, placebo-controlled biomarker trial, 38 early-stage breast cancer patients received 11 days of perioperative blockade of catecholamines and PGs with propranolol and etodolac [325]. Excised tumors and sequential blood samples were assessed to determine the effects of excess perioperative release of catecholamines, PGs and prometastatic biomarkers [325]. Treatments were well tolerated and significantly decreased EMT, reduced the activity of prometastatic/proinflammatory transcription factors GATA binding protein (GATA)-1, GATA-2, EGR3 and STAT3, decreased tumor-infiltrating monocytes, increased tumor-infiltrating B cells, abrogated presurgical increases in serum IL-6 and C-reactive protein (CRP) levels, abrogated perioperative declines in stimulated IL-12 and IFNγ, mobilized CD16^−^ “classical” monocytes, and enhanced expression of CD11a in circulating natural killer cells [325]. Similar results were obtained in a phase II double-blind placebo-controlled trial of colorectal cancer [326].

Adjuvant chemotherapy after surgery is administered for the intended effect of eliminating micrometastases but may have different impacts on micrometastases that are slowly proliferating compared to the dormant ones. Patients with metastatic breast cancer who have a synchronized onset of growth among micrometastases, as defined by low variance among the sizes of metastatic lesions, have shorter times to recurrence after surgery, an effect counteracted by adjuvant systemic therapy that may also antagonize the effects of systemic growth signals caused by surgery [327]. 

Data from another scenario in a pancreatic cancer murine model support the role of inflammation in perturbing micrometastases [328]. The study demonstrates that abdominal surgery and resection of pancreatic ductal carcinoma induces inflammation in the liver, which converts micrometastasis-suppressing hepatic stellate cells into hepatic myofibroblasts, which in turn promote the outgrowth of micrometastases in tumor necrosis factor (TNF)-related apoptosis-inducing ligand-receptor 2 (TRAIL-R2)- and CXCL-8/IL-8-dependent manner [328]. A retrospective study of 327 patients undergoing mastectomy for breast cancer demonstrated that patients given a non-steroidal anti-inflammatory drug had a significantly superior disease-free survival in the first 5 years after surgery than patients who did not receive the anti-inflammatory drug, with a five-fold reduction in early relapse events [329]. A meta-analysis of 49 publications reported a slightly protective effect of non-steroidal anti-inflammatory drugs, aspirin, and COX-2 inhibitors for hormone receptor-positive breast cancer [330]. A study of more than 24 thousand patients from a health insurance database used a Cox proportional hazard model to demonstrate an association between propranolol use for over 6 months and a significantly lower risk of developing several solid tumors compared to patients who did not take propranolol [331]. The study had over a 12-year follow-up and demonstrated that the effect was greatest when the exposure duration exceeded one thousand days [331]. These studies, while not addressing BM or other micrometastases, recurrence or breast cancer specifically, provide a context for suppressing inflammation and catecholamine signaling in cancer promotion or progression. Hence, the immediate perioperative period encompasses driving forces in two directions: ones that suppress micrometastases and ones that awaken them. It is important to investigate the clinical implications of interventions that tilt the balance to maintaining dormancy [332].

### 3.6. Stress, Neuradrenergic Stimuli and Depression

The neuroendocrine activation of brain cytokines through stress influences events in the bone marrow microenvironment through a dual system. The first effect translates to psychological stress to disrupt bone marrow niche homeostasis through the secretion of systemic glucocorticoids and catecholamines that facilitate an inflammatory response, downregulate inhibitory receptors on microglia, and prime inflammation mediated by monocytes and macrophages to promote departure from dormancy [333]. The second effect of neuroendocrine activation is direct and is mediated through sympathetic nerve signaling in the stem cell niche [333]. In the bone marrow, sympathetic nerve fibers represent a critical component of the niche, forming rings around osteoblasts and osteocytes, and, in fact, have more sensory and sympathetic fibers in total than the mineralized bone or the periosteum [334].

The differentiation, maturation and proliferation of all levels of hematopoietic precursors, stromal cells, macrophages and Thy1/2^+^ cells are under the control of centrally directed monoaminergic regulation [335]. Adrenaline and noradrenaline dramatically enhance IL-33 expression in dendritic cells upon lipopolysaccharide stimulation, mediated by AR-β2 through protein kinase A (PKA) and cyclic AMP (cAMP), suggesting a mechanism contributing to the activation of Thy2 cells [336]. Erythropoietic differentiation is more sensitive to serotonergic influences via specific receptors, regardless of whether it is in the setting of normal hematopoiesis, hyperplasia, myelosuppression or dysregulated progenitor differentiation, whereas myelopoiesis is more sensitive to central catecholamines [335]. 

Chronic psychological stress has immunosuppressive functions through the accumulation of MDSCs, which can suppress inflammation [337] and promote tumor progression [214]. In a murine model, dual stimulation with β-agonist infusion and chronic psychological stress caused the accumulation of CD11b^+^ gamma response (Gr1)^+^ lymphocyte antigen (Ly)6G^+^Ly6C^low^ immature neutrophils, potentially through cyclooxygenase 2 (COX-2)- PGE2 signaling, which inhibits cytokine release by macrophages and T-cell responsiveness but does not directly cause MDSCs accumulation [338].

Processes linked to dormancy reactivation include stressful events, which can cause the release of neural factors in the niche [339], sympathetic signaling [206] and catecholamines that induce tumor growth directly and indirectly through non-autonomous mechanisms [339]. Interestingly, although discussed as a potential cause of dormancy recurrence in case reports, a specific investigation of breast cancer recurrence after traumatic events in patients from a clinical trial database concluded that it did not have a greater than random association under the design and circumstances of that study [340].

Direct mechanisms of tumor stimulation by stress include suppression of anoikis, suppression of apoptosis through induction of B-cell lymphoma 2 (BCL-2), B-cell lymphoma-extra large (BCL-xL), myeloid leukemia cell differentiation (MCL), suppression of the pro-apoptotic Bcl-2-associated death promoter (BAD), induction of phosphorylation of FAK, activation of AR-β2 signaling and acceleration of tumor progression in a variety of animal models [339]. Non-autonomous effects through multiple forms of α- and β-ARs induce angiogenesis, modulate adhesion molecule expression on stromal components and remodel stromal proteins that promote cancer proliferation, among others (expertly reviewed by Hanns et al., 2019 [339]. Chronic psychological stress in animal models also disturbs long bone growth during development, disrupting processes that may remain relevant in the endosteum of adult bone with respect to interactions with the stem cell niche [341]. In fact, it disrupts normal hematopoiesis in the niche [342]. Catecholaminergic fibers in the BM of mice are necessary for HSC mobilization at a steady state and under stress, and mobilization by the nervous system is mediated through SDF-1/CXCR4, proteolytic enzymes, urokinase plasminogen activator (uPA) and bone remodeling [343,344]. The erythropoietic system is more sensitive to serotonergic influences, regardless of the pathologic conditions of hyperplasia, myelosuppression or dysregulation of progenitor differentiation, whereas myelopoiesis is more sensitive to central catecholamines [335]. 

Norepinephrine may play a role in the reawakening of prostate cancer micrometastases. In vitro, norepinephrine stimulates prostate cancer cell proliferation through AR-β2 [206]. In the bone marrow niche, norepinephrine downregulates the secretion of dormancy-inducing GAS6 in osteoblasts through an indirect mechanism [206]. An unbiased bioinformatics pipeline demonstrated that norepinephrine-mediated sympathetic signaling through ATF1, RAR and E2F nodes downregulated GAS and was associated with dormant prostate cancer recurrence and cell cycle reentry in the bone marrow [345].

Depression induces the secretion of serotonin or 5-hydroxytryptophan (5-HT) by duodenal enterochromaffin cells, its primary site of synthesis, and produces a defect in its uptake by platelets and neurons, causing increased levels of circulating 5-HT [207]. Breast cancer cells express four types of 5-HT receptors, and their binding to 5-HT induces PTHrP through RUNX2, inhibits osteoblast maturation and activates osteoclasts through the RANKL pathway [207]. In a murine model of chronic mild stress, the resulting depression promoted bone metastases from an injection of a triple-negative human breast cancer cell line [207]. It is likely that high circulating 5-HT levels in depression may potentially induce dormant breast cancer cells in the niche to synthesize PTHrP and activate osteoclasts, resulting in reawakening signals from bone resorption. A meta-analysis of 17 studies of over 280 thousand breast cancer patients demonstrated that depression is associated with breast cancer recurrence, all-cause mortality and cancer-specific mortality [346]. The study demonstrated also that anxiety is associated with breast cancer recurrence and all-cause mortality [346]. The risk factors for clinically diagnosed depression and anxiety demonstrated in the study were females who were in the under-60-year category, with a shorter follow-up duration and worse prognosis [346]. 

**Table 5:** Surgery, angiogenesis, inflammation and catecholamine effects on dormant BC cells.

	Mechanism	Vehicle and Function	Signaling	References
Angiogenesis				
	Endothelial cell stimulation	Endothelial cell tips	TGF-β1, periostin, Gli-1, Wnt	[159,160,162,311]
	Translocation of dormant BC cells from endosteal to the endothelial niche		L1CAM, YAP, MRTF, integrin β1, L1CAM	[307]
	Intermittent hypoxia	Endothelial sprouting	Hif-1 and -2, angiogenic factors	[308]
Surgery	Primary tumors (or local recurrences) secrete metastasis-stimulating factors	IL-6, IL-8, VEGF, EGF, PDGF-AA, MIF, SerpinE1, and M-CSF	Disseminated BC cell growth signals	[315]
	Surgically induced inflammatory responses	- eliminate dendritic cells - eliminate activation of cytotoxic T-cells tumor-directed responsible - macrophage, NK cell, monocytes dysfunction	eliminate immune suppression contributing to dormancy	[204,319,320]
	Surgically-induced stress	- Generation of catecholamines, β-adrenergic agonists, prostaglandins - EMT	- Elevated perioperative IL-6, IL-8, NF-kB, CRP - reduced IRF1 - promotes growth of micrometastases - increases GATA-1 GATA-2, EGR3, STAT3 activity - increased tumor-infiltrating monocytes - decreased tumor-infiltrating B cells - perioperative decline in stimulated IL-12 - perioperative decline in IFNγ, mobilization of CD16^−^ monocytes - decreased expression of CD11a on circulating NK cells	[321,322,325,326]
Stress, noradrenergic stimuli, depression	stressful events, sympathetic signaling, catecholamines	- systemic glucocorticoids and catecholamines - inflammatory response - direct activation of sympathetic nerve fiber signaling around osteoblasts and osteocytes the stem cell niche - suppression of anoikis and apoptosis - angiogenesis, stromal adhesion molecule expression - stromal protein remodeling	- downregulated inhibitory receptors on microglia - inflammation mediated by monocytes and macrophages - differentiation, maturation, proliferation of stromal cells, macrophages, Thy1/2^+^ cells - enhanced IL-33 expression by dendritic cells upon lipopolysaccharide stimulation mediated by - AR-β2, PKA, cAMP - promote dormant cell reactivation - activation of BCL-2, BCL-xL, MCL, pFAK, AR-β2 signaling - suppression of BAD - downregulates GAS6 in osteoblasts - ATF1, RAR, E2F	[206,333,334,335,336,339,345]
	Chronic psychological stress	- immunosuppressive functions	accumulation of MDSCs - suppressed inflammation - accumulation of CD11b^+^Gr1^+^ Ly6G^+^Ly6C^low^ immature neutrophils - COX-2- PGE-2 - inhibits macrophages cytokine release Inhibits T-cell responsiveness - tumor promotion	[214,337,338]
	Depression and anxiety	circulating 5-HT bind BC cell receptors	- 5-HT Uptake by platelets and neurons - PTHRP production by BC via RUNX2 - inhibits osteoid maturation - activates osteoclasts through RANKL	[207,346]

## 4. Potential Therapeutic Approaches

The tripartite approach to targeting dormant quiescent DTCs, that of either killing them, maintaining them in a continuously dormant state or reawakening them to render them susceptible to therapy, has been discussed for many years and articulated by many investigators [347,348,349]. Most hypothesis-driven investigations focus on the first two approaches, as awakening dormant cells to make them susceptible to killing generates a set of circumstances with more anticipated complexities [350].

However, the current state of understanding in the field is primarily based on approaches and hypotheses generated in vitro using cell lines or animal models. Many investigations suggest that specific in vitro and in vivo observations may have potential efficacy in eradicating dormant cells or maintaining their dormant status, but clinical trials testing these hypotheses are relatively rare. In addition, preclinical models have been used to investigate a variety of solid tumor types, hematopoietic malignancies and metastatic sites. Hence, most hypotheses for treating dormant breast cancer cells in the bone marrow are generated by constructing analogies. The following discussion focuses on examples of preclinical approaches that suggest potentially relevant outcomes for breast cancer cells in the bone marrow and several approaches that have been or are being investigated in clinical trials. 

Approaches that have yielded promising data in preclinical models include the use of alkylating agents to eliminate non-cycling residual primary BRCA^−^/p53^−^ breast cancer cells in vivo [351], inhibition of Src family kinases (SFK), Src and MEK1/2 signaling [223,352], treatment with BMP7 [211] and VCAM-1 and integrin α4 antibodies [219] to suppress metastatic breast cancer outgrowth, inhibition of ERK and p38 [119] and PI3K/Akt [98,119] to diminish dormant ER^+^ BC cell survival in vitro. Targeting autophagy with hydroxychloroquine demonstrated significant decreases in the detectable autophagy-related 7 (ATG7) gene, accumulation of damaged mitochondria and reactive oxygen species (ROS), resulting in apoptosis and decreased survival of dormant cells [201]. Blocking autophagy with chloroquine diphosphate also restores estrogen sensitivity in breast cancer cells [353]. Other in vitro approaches have investigated the use of polymers and nanoparticles to target dormant cancer cells [348,354].

The osteogenic niche acts as a calcium reservoir for dormant cancer cells [53]. Ca^++^ is transported through gap junctions, and together with mTOR signaling, can promote metastasis progression, with the blocking of gap junctions delaying bone colonization [53]. Danusertib, a small molecule pan-aurora kinase inhibitor alone, or combined with the FDA-approved drugs everolimus and arsenic trioxide, inhibits this signaling, downregulates gap junctions in cancer cells specifically, diminishes micrometastases without affecting primary tumors, prevents colonization, and demonstrates that these drugs may be candidates for eliminating breast cancer bone marrow micrometastases in clinical trials [53]. This approach can be used alone or in combination with adjuvant chemotherapy. 

Regulation of redox signaling to prevent dormant cells from reawakening has been tried in preclinical models using antioxidants or conventional therapies inducing excess oxidative stress [140]. However, since redox regulatory mechanisms are highly dependent on circumstances, on the dose and on time-specific redox levels, a more nuanced approach must be adopted [140].

Investigations have demonstrated roles for integrin α5β1 binding to fibronectin in establishing the premetastatic niche [81], the dormant phenotype, anchorage [98], signaling [119,120] and resistance to treatment [98,119]. Integrins are necessary for maintaining dormancy through the obligate formation of organized fibrillary fibronectin via integrin αvβ3 and α5β1 adhesion, generation of ROCK-mediated tension and TGFβ2 stimulation [103]. The data also suggest that integrin αvβ3 is involved in the colonization of the osteoblast niche by breast cancer cells and its inhibition may be a potential approach to prevent cancer cell regrowth in the bone marrow [355,356]. RUNX2 promotes the attraction and adhesion of breast cancer cells to the bone and confers cancer cell survival and bone colonization advantages in mice through its transcriptional target integrin α5, establishing integrin α5 as a potential target for preventing bone colonization [35]. RUNX2 positively correlates with bone metastasis in patients with lymph node-negative breast cancer [35].

Integrin α5 is highly expressed in bone metastases compared to lung, liver, or brain metastases, and its expression in primary tumors correlates with the presence of DTCs in bone marrow aspirates from early-stage breast cancer patients [357]. Its expression is both necessary and sufficient for tumor cell adhesion to fibronectin, migration, and survival in vitro, tumor cell colonization of the bone marrow and formation of osteolytic lesions in vivo in mice [357]. Pharmacological inhibition with the humanized monoclonal α5β1 antibody volociximab (M200) inhibits tumor cell migration and survival in vitro and colonization of the bone marrow as well as osteoclast-mediated bone resorption, as integrin α5 is also expressed on osteoclasts [357]. Disruption of dormant cancer cell survival and recurrence can be achieved by blocking integrin β1 signaling with antibodies in the fibrotic marrow [223,358], blocking myosin light chain kinase (MLCK) activation by integrin β1 to prevent the formation of F-actin and proliferative growth [104], blocking fibronectin attachment [98] or downregulating integrin β1 expression with flavopiridol to decrease attachment and survival of dormant ER^+^ BC clones [119]. The inhibition of integrin β1- and αvβ3-mediated interactions between disseminated breast cancer cells and the perivascular niche, mediated partly by endothelial-derived vWF and VCAM-1, sensitizes the cancer cells to chemotherapy, without inducing proliferation or exacerbating toxicity, and prevents bone metastasis [8].

The apparently obligatory role of integrins in the generation and maintenance of breast cancer dormancy in the bone marrow has become the basis for arguments to investigate the therapeutic testing of blocking antibodies or peptides to relevant integrins in conjunction with adjuvant chemotherapy or other biologicals to eliminate micrometastases [358,359]. Treatment of mice with Ac-PHSCN-NH2 (ATN-161), a 5-mer-capped peptide derived from the synergistic region of fibronectin that blocks binding to integrin α5β1 and αvβ3, resulted in significant decreases in pERK, microvessel density, tumor cell proliferation in vivo, a significant dose-dependent decrease in tumor volume and skeletal and soft tissue metastases [360]. A phase I clinical trial with ATN-161 demonstrated tolerability and safety, achievement of serum levels consistent with those found to have efficacy in animals, and also stability in metastatic disease in a significant fraction of patients [361]. 

Bisphosphonates inhibit the effects of osteoclast bone resorption and have had a long-known impact on suppressing cancer progression in the bone marrow. A clinical trial was conducted to determine the effects of zolendronate administered every four weeks on the presence of disseminated breast cancer cells in bone marrow aspirates of patients who underwent surgery and adjuvant chemotherapy, and the effects on progression-free survival [362]. The data demonstrated a significant reduction in the detection of DTCs after 6 months of zolendronate in a repeat bone marrow aspiration compared to control patients who did not receive zolendronate after adjuvant therapy, and reduced recurrence-free survival in patients with persistent isolated tumor cells in the aspirates [362]. Another clinical trial is ongoing to determine the different effects of bisphosphonates zolendronate, clodronate or ibandronate on bone metastatic recurrence in breast cancer [363].

In an animal model, ivermectin, an antiparasitic drug used for treating river blindness, inhibited NETs by inhibiting myeloid cell infiltration [364]. Ivermectin suppresses the release of granulocyte extracellular DNA and proteins by targeting the lytic cell death modality-inducing pyroptosis or inflammatory cell death-driving factor gasdermin, inhibiting the oligomerization required for NET formation, and significantly suppressing melanoma lung metastasis [364]. The release of extracellular DNA and proteins, a process mediated by the enzyme peptidyl arginine deiminase 4 (PADI4) and translocation of elastase to the nucleus, activates tumor-associated fibroblasts in another model, that of pancreatic stellate cells, and inhibits stromal activation that promotes cancer cell proliferation and metastasis [365]. BM transplanted from PADI4-deficient mice into genetically engineered oncogene-driven tumor-forming mice limits invasive tumor formation [365]. Targeting NETs and PADI4 seem to be potential strategies in solid tumor therapy, but the approach has not reached the level of clinical trials yet in preventing metastasis reawakening and needs to be investigated in preclinical models [365]. 

Proposals to use epigenetic modifications with HDAC inhibitors or DNA demethylating agents to induce or maintain dormancy through activation of RARβ and CDKN1A (p21^Waf1^) have been presented based on in vitro observations on their effects of inducing cell cycle inhibition in other malignancies [176]. In vitro treatment of ER^+^ and triple-negative breast cancer or other epithelial and hematologic malignant primary cells and cell lines with the DNA methylation inhibitors decitabine and azacitidine at physiologic, non-toxic nanomolar doses produces antitumor “memory” responses rather than acute toxic antitumor effects, without affecting hematopoietic progenitors [366]. The “memory” effects denote that AZA treatments at doses that do not kill cells according to the time course observed with cytotoxins induce sustained effects on gene expression that result in new phenotypes and antitumor effects that last long after drug withdrawal. These effects include sustained direct and indirect activation of new antitumor regulatory pathways, including apoptosis, diminished cell cycling, inhibition of cancer stem-like cells, sustained decreases in genomewide promoter DNA methylation and induction of gene re-expression [366]. Additional cell functions that are affected include upregulation of lineage commitment, epithelial-type keratin re-expression, blocking of cell cycle entry and progression, modulation of signal transduction, regulation of cytoskeletal remodeling, anaerobic glycolysis, enhanced immune response, antigen presentation and anti-inflammatory effects [366]. Epigenetic modulation also affects the growth of primary and secondary transplanted xenografts, underscoring their chronic effects on the cells’ phenotypic characteristics that persist after drug removal [366]. The development of selective HDAC inhibitors has shown some antitumor successes in the advanced or recurrent stages of several cancers in clinical trials [367]. In addition, some selective inhibitors have demonstrated preclinical in vivo effectiveness in suppressing the metastatic ability of transplanted breast cancer cell lines [368]. Other potential candidates, such as curcumin, have shown some in vitro mechanistic efficacy in inducing tumor suppressor genes, such as deleted in liver cancer (DLC)1, but remain to be tested in models [369]. An agonist of NR2F1 prevents lung metastases and induces dormancy in head and neck squamous cell carcinoma metastases in a mouse model [370].

Immune therapy for dormant micrometastases has been proposed based on preclinical and clinical evidence [371]. Randomized phase II clinical trials of patients with node-positive or high-risk node-negative Her2^+^ BC after definitive therapy and no clinical evidence of disease demonstrated a potential clinical benefit in survival [372,373]. Patients received vaccines with the Her2 peptide GP2, which elicits a CD8^+^ response against the HER2 antigen, and granulocyte-macrophage colony-stimulating factor (GM-CSF) [372], or with the Her2 peptide AE37, which primarily elicits a CD4^+^ response against Her2, with and without GP2, or GP2 alone [373], with some of the patient subsets demonstrating improved survival. Cancer cells that are truly dormant after chemotherapy and negative for Ki67 are susceptible to immune modulation, whereas cells that are low for Ki67 are not [374]. Based on these observations, it has been proposed that immunotherapy should be administered after therapeutic conditioning to suppress tumor immunoediting that permits escape from immunotherapy or in combination with targeted therapies [371]. 

Suppressing immune-mediated reawakening has been addressed in a model using a natural compound, the flavinoid kaempferol, found in fruits and vegetables [375]. Kaempferol significantly inhibits the NET formation and metastatic tumor formation in the lungs in an animal model and suppresses ROS production in mouse BM-derived neutrophils [375]. 

Another key intervention in the peritherapeutic period involves control of the inflammatory responses, tumor shedding, pro-angiogenic and growth factor-generating effects of cancer surgery that stimulate micrometastases established prior to the diagnosis of the primary tumor, initiation of new metastases and disrupting immune control over them [376]. These investigators hypothesize that certain types of immunotherapy, such as abrogation of stress-inflammatory responses, could be initiated before the administration of adjuvant therapy to minimize the pro-awakening effects of therapy [376]. As noted earlier, anxiety in the perioperative period activates the sympathetic nervous system and releases catecholamines during cancer surgery and inflammation to facilitate prometastatic processes [377]. Clinical evidence shows that perioperative β-adrenoreceptor blockade and COX-2 inhibition are safe, feasible and able to reduce the prometastatic process and cancer recurrence [377]. In addition, psychophysiological approaches may substitute for pharmacological approaches to reduce catecholamine effects [377]. 

In a rat model, preoperative behavioral stressors reduce synthetic TLR-4-induced IL-12 levels and resistance to syngeneic experimental breast cancer metastases, and enhance the deleterious effects of laparotomy on metastasis [378]. These effects are abrogated by treatment with the glucocorticoid receptor antagonist RU-486 [378]. In addition, the deleterious effects of stress and surgery on post-operative resistance to experimental metastasis are eliminated by antagonizing the impact of glucocorticoids before surgery, activating pre-operative anti-metastatic immunity perioperatively, and blocking operative and postoperative adrenergic and prostanoid responses [378].

Other approaches, those of rendering dormant micrometastases susceptible to the adjuvant chemotherapy already being administered for the very purpose of eliminating them at the time of initial treatment for localized breast cancer, are likely to be highly compelling [359]. Blocking integrin binding to the microenvironment or the survival, EMT and stem cell signaling programs initiated by interactions with the microenvironment may render cells susceptible to cytotoxic therapy and significantly diminish dormant cell numbers or their ability to re-enter the cell cycle. 

Other clinical trials have taken various approaches to keep disseminated cells in their dormant state, including testing the anti-proliferative, anti-inflammatory, and antioxidant properties of muscadine grape products [379] or green tea catechin extract polyphenon E [380] to decrease systemic inflammation. A prospective, open-label, randomized, cross-over, pilot study to determine if reprogramming therapy in patients with recurrent PCa only based on rising PSAs maintains dormancy has been completed. The primary objectives were to compare the disease progression-free rate at the end of 12 weeks between 5-AZA+ATRA and no therapy, and to assess the safety of the 5-AZA and ATRA combination [381].

In summary, most treatment approaches have been tested in preclinical settings and are either directed at eliminating dormant cells or keeping them dormant. Preclinical studies have focused on eliminating dormant cells using alkylating agents, inhibitors of metastatic outgrowth signaling pathways, autophagy inhibitors, polymers and nanoparticles, regulators of redox signaling, inhibitors of adhesion molecule-initiated signaling, including survival signaling that provides resistance to adjuvant therapy, bisphosphonates and glucocorticoid receptor antagonists to reduce perioperative stress. Preclinical studies to maintain dormancy have used the suppression of NET formation by ivermectin, epigenetic modifications, curcumin and NR2F1 agonists, and suppression of immune-mediated reawakening. Clinical trials have tested immunotherapy for dormant cancer cells, perioperative β-adrenoreceptor blockade, COX-2 inhibition, psychophysiological approaches to reduce the prometastatic process and cancer reawakening, and antioxidants and differentiating agents to maintain dormancy.

Novel trial designs to determine the effects of therapy directed at dormant micrometastases have been proposed, where the endpoint would be the assessment of the time to a later recurrence after an initial recurrence is treated [349]. Clinical interventions to eliminate micrometastases either by direct treatment or with treatment to sensitize them to adjuvant chemotherapy are still in the aspirational phase in medical oncology. Adjuvant chemotherapy, hormonal or biotherapy eliminates some dormant disseminated cells and decreases their chances of recurring, but such treatment only shifts the survival curve, as does the maintenance therapy for hormone ablation for ten years in hormone-sensitive breast cancer. These approaches will continue to be tested in clinical trials, and perhaps eventually, therapies will be able to eliminate disseminated breast cancer cells.

## 5. Conclusions

Breast cancer cells disseminate before primary tumors can be diagnosed and populate the bone marrow with sparse dormant therapy-resistant micrometastases. These cells provide a pool of cells that recur and cause incurable metastatic disease steadily for more than two decades. Dormant cells in the BM are rendered and remain dormant in the HSC niche by interactions with endosteal, endothelial and immune niches, as well as with physicochemical local conditions and intrinsic signaling. Reawakening occurs stochastically at different rates in hormone-independent and hormone-dependent BC types. Specific circumstances driving recurrence cannot be predicted, but predisposing factors stimulating recurrence have been investigated, including inflammation, aging, loss of MSC quality characterized by loss of FGF-2 production, lack of regenerative function, redirected adipogenic differentiation, estrogen deprivation, stromal injury, hypercoagulable states, angiogenic bursts, surgery-induced catecholamine production and inflammation, noradrenergic stress, and depression. Most of these factors do not occur independently and are difficult to identify as specific causes of recurrence in individual circumstances. A wide variety of therapeutic interventions to eliminate dormant micrometastases, either with targeted therapies, sensitizing dual approaches or interventions intended to maintain them in a dormant state indefinitely, have been investigated in preclinical models, both in vitro and in vivo, and some have been promising. The field has begun to progress to clinical trial investigations of some of the more promising approaches, with some modicum of success. The volume and diversity of innovative approaches with potential preclinical benefits will ensure their expansion and the eventual success of some of them in clinical trials.
